# Comparative Transcriptomic Analysis of Two Actinorhizal Plants and the Legume *Medicago* *truncatula* Supports the Homology of Root Nodule Symbioses and Is Congruent With a Two-Step Process of Evolution in the Nitrogen-Fixing Clade of Angiosperms

**DOI:** 10.3389/fpls.2018.01256

**Published:** 2018-10-08

**Authors:** Kai Battenberg, Daniel Potter, Christine A. Tabuloc, Joanna C. Chiu, Alison M. Berry

**Affiliations:** ^1^Department of Plant Sciences, University of California, Davis, Davis, CA, United States; ^2^Department of Entomology and Nematology, University of California, Davis, Davis, CA, United States

**Keywords:** actinorhizal plants, evolution, nitrogen fixation, nitrogen-fixing clade, orthology, root nodule symbiosis, transcriptomics

## Abstract

Root nodule symbiosis (RNS) is a symbiotic interaction established between angiosperm hosts and nitrogen-fixing soil bacteria in specialized organs called root nodules. The host plants provide photosynthate and the microsymbionts supply fixed nitrogen. The origin of RNS represents a major evolutionary event in the angiosperms, and understanding the genetic underpinnings of this event is of major economic and agricultural importance. Plants that engage in RNS are restricted to a single angiosperm clade known as the nitrogen-fixing clade (NFC), yet occur in multiple lineages scattered within the NFC. It has been postulated that RNS evolved in two steps: a gain-of-predisposition event occurring at the base of the NFC, followed by a gain-of-function event in each host plant lineage. Here, we first explore the premise that RNS has evolved from a single common background, and then we explore whether a two-step process better explains the evolutionary origin of RNS than either a single-step process, or multiple origins. We assembled the transcriptomes of root and nodule of two actinorhizal plants, *Ceanothus* *thyrsiflorus* and *Datisca* *glomerata*. Together with the corresponding published transcriptomes of the model legume *Medicago* *truncatula*, the gene expression patterns in roots and nodules were compared across the three lineages. We found that orthologs of many genes essential for RNS in the model legumes are expressed in all three lineages, and that the overall nodule gene expression patterns were more similar to each other than expected by random chance, a finding that supports a common evolutionary background for RNS shared by the three lineages. Moreover, phylogenetic analyses suggested that a substantial portion of the genes experiencing selection pressure changes at the base of the NFC also experienced additional changes at the base of each host plant lineage. Our results (1) support the occurrence of an event that led to RNS at the base of the NFC, and (2) suggest a subsequent change in each lineage, most consistent with a two-step origin of RNS. Among several conserved functions identified, strigolactone-related genes were down-regulated in nodules of all three species, suggesting a shared function similar to that shown for arbuscular mycorrhizal symbioses.

## Introduction

Root nodule symbiosis is a symbiotic interaction established between certain groups of angiosperm hosts and nitrogen-fixing soil bacteria that are housed in specialized organs called root nodules. The host plants provide photosynthate to their microsymbionts, and in turn the microsymbionts provide fixed nitrogen to their host plants.

This symbiotic relationship enables host species to thrive in nutrient-poor soils, and thus these RNS hosts play a major role in terrestrial ecosystems as pioneer plants (Chapin et al., 1994). Moreover, legumes play key roles in agriculture, where plant-based biological nitrogen fixation accounts for as much as 10% of the total nitrogen fixed in the world (Herridge et al., 2008; Fowler et al., 2013). Thus, understanding the genetic underpinnings of the origin of RNS not only provides insight into a major biological event in the evolution of angiosperms, but is also of major economic and agricultural importance.

RNS occurs in ten families of angiosperms within four orders: Fabales, Rosales, Cucurbitales, and Fagales. Molecular phylogenetic studies have revealed that these four orders, which were previously considered to be distantly related within the angiosperms (Cronquist, 1988), together form a clade known as the nitrogen-fixing clade (NFC) (Soltis et al., 1995). Within each of the four orders, RNS occurs in a subset of the families, which are phylogenetically scattered within each order (Swensen, 1996), and within each family, RNS is restricted to a subset of the genera.

There are several possible hypotheses regarding the evolutionary origin of RNS that can explain this restricted (found only among orders of the NFC) yet scattered (found only in some families and genera of the NFC) distribution of RNS hosts (Doyle, 2011). The single-origin hypothesis proposes that the capability of forming nitrogen-fixing root nodules evolved once in the MRCA of the NFC and was subsequently lost multiple times independently in the currently non-fixing lineages. The multiple-origin hypothesis proposes that the evolution of RNS occurred independently at least six and as many as ten times (Doyle, 2011). The two-step hypothesis postulates that a predisposition for, i.e., propensity to subsequently gain, RNS was first gained at the base of the NFC, which was then followed by a gain of function that occurred independently in the aforementioned six to ten different lineages (Soltis et al., 1995; Swensen, 1996; Werner et al., 2014).

The two-step hypothesis has been supported by phylogenetic analysis based on the distribution of RNS hosts within the NFC (Werner et al., 2014). The hypothesis can parsimoniously explain the restricted yet scattered phylogenetic distribution of RNS hosts, but raises the question, what was the genetic basis of the “predisposition” to RNS?

In addition to the phylogenetic evidence, several shared cellular, molecular, and genetic characteristics of RNS hosts in different lineages are consistent with a common evolutionary predisposition to RNS: (1) all RNSs result in a stable accommodation of the microsymbiont within the host cells (Pawlowski and Demchenko, 2012); (2) homologs of many essential genes required for RNS in the model legumes *Medicago* *truncatula* and *Lotus* *japonicus* have been shown to be expressed in the nodules of non-legume RNS hosts (Hocher et al., 2011; Demina et al., 2013); (3) calcium oscillation, an early host physiological response, is induced during initiation of RNS in both legume and non-legume hosts (Granqvist et al., 2015).

Multiple lineages sharing these aforementioned series of characters support the common descent of RNS across multiple lineages (i.e., supports single-origin and two-step hypotheses over multiple-origin hypothesis), but some morphological, cellular, and molecular characteristics are clearly distinct in different lineages of RNS hosts (Pawlowski and Demchenko, 2012), which could favor a multiple-origin hypothesis. Most notably, RNS hosts in two families (Fabaceae and Cannabaceae) associate with rhizobia as their microsymbiont, while the hosts in the remaining eight families associate with members of the actinobacterial genus *Frankia*. These eight families are collectively called the actinorhizal plants. Most *Frankia* genomes lack the genes coding for Nod factor, the signaling molecule responsible for the initiation of RNS in the model legumes (Oldroyd, 2013), but homologs for the *nodABC* genes have been identified in some groups of *Frankia* (Persson et al., 2015; Nguyen et al., 2016). Similarly, some legume hosts can be nodulated by rhizobia without the Nod factor signaling pathway (Okazaki et al., 2015). Therefore, a range of different mechanisms for initiating RNS must exist among the legumes and the actinorhizal plants.

Transcriptomes generated via RNA-seq represent a powerful source of data that can provide a comprehensive set of characters. In the present study, we have used evidence from comparative transcriptomics and molecular evolutionary analyses to test the competing hypotheses for the evolutionary origin(s) of RNS. To this end, we assembled the root nodule and root transcriptomes of two actinorhizal plant species, *Ceanothus* *thyrsiflorus* (Rhamnaceae, Rosales) and *Datisca* *glomerata* (Datiscaceae, Cucurbitales) and compared them to published transcriptomes of *M.* *truncatula* (Fabaceae, Fabales) (Roux et al., 2014). We conducted differential gene expression analysis between nodules and roots to determine a set of genes that are root- or nodule-enhanced for each species. Then, to allow interspecific comparisons, phylogeny-based orthology prediction was conducted across the three species and other taxa that are either members or close outgroups of the NFC.

We first explored if RNS has evolved from a single common evolutionary event that occurred at the base of the NFC, regardless of whether this event gave rise to the function or the predisposition of RNS. To test the homology of RNS in the three species, we first focused on the presence/absence of orthologs in *C.* *thyrsiflorus* and *D.* *glomerata* for 19 genes required for the initiation and development of root nodules in the model legumes *M.* *truncatula* or *L.* *japonicus*, because both the single-origin hypothesis and the two-step hypothesis would require that at least some of the orthologs of genes required for nodulation would be shared among all RNS hosts. Determining orthology is an improvement with respect to previous studies that have identified homologs of genes required for RNS in legumes in several actinorhizal plants (Hocher et al., 2011; Demina et al., 2013), as orthologs are a subset of homologs that are most likely to be functionally equivalent according to ortholog conjecture (Koonin, 2005; Altenhoff et al., 2012). A recent study has taken a similar approach, comparing nodule gene expression in *Parasponia* spp. the only non-legume host that can establish RNS with rhizobia, to *M.* *truncatula* (van Velzen et al., 2018).

Second, we compared the overall expression patterns of orthologous genes across the three species between roots and nodules. Our assumption was that a significant degree of similarity across the three species, which are known to belong to three different lineages of RNS hosts (Doyle, 2011), is not expected if they had completely independent origins of RNS. Thus, sharing a significant degree of similarity would refute the multiple-origin hypothesis, and indicate a single common ancestor for RNS. Gene expression analysis and orthology predictions allowed us to identify a set of genes that showed a consistent pattern of differential expression across the three species, which we designated as the core set of genes for RNS.

Then, we have further explored which of the three competing hypotheses best explains the origin of RNS, particularly whether a single-origin or a two-step process better explains the evolutionary basis of RNS. For this, we employed a model-based phylogenetic test. We focused on the fact that the three hypotheses each assume different timing(s) for the gain-of-predisposition or gain-of-function event leading to RNS in the evolutionary history of the NFC. We assumed that gain of a new function would result in a change in selection pressure on that gene, which should be reflected in the average ratio of non-synonymous to synonymous mutations (*dN*/*dS*) (Hurst, 2002) in the coding regions of the gene. Thus, for each set of orthologs, we tested when (if ever) each member gained a new function by calculating *dN*/*dS* on key branches of their respective phylogenetic trees.

## Results

### Transcriptome Assembly and Completeness

Illumina HiSeq platform generated a total of 376,256,549 and 303,175,236 paired-end reads (150 bp) for *C.* *thyrsiflorus* and *D.* *glomerata*, respectively. After cleaning, >97% (342,814,393 reads) and >91% (296,864,053 reads) of these pairs were kept for the transcriptome assembly (**Supplementary Table S1**, NCBI BioProject ID: PRJNA422680). Trinity (Grabherr et al., 2011) generated root + nodule transcriptomes for *C.* *thyrsiflorus* and *D.* *glomerata* with 675,696 transcripts (480,254 genes) and 444,766 transcripts (309,847 genes), respectively. After curating (three screenings and collapsing of alleles), the cleaned transcriptome of *C.* *thyrsiflorus* and *D.* *glomerata* consisted of 15,245 and 15,448 genes, respectively (**Supplementary Table S2**, NCBI BioProject ID: PRJNA422680). In both transcriptomes, N50, the length of the shortest transcript that covers 50% of the transcriptome, was >2.1 kb, average was >1.8 kb, and median was >1.6 kb. Over 76% and 81% of the cleaned reads mapped onto the cleaned transcriptomes of *C.* *thyrsiflorus* and *D.* *glomerata*, respectively (**Supplementary Table S2**).

Within each of the three root + nodule transcriptomes (*C.* *thyrsiflorus, D.* *glomerata*, and *M.* *truncatula*), BUSCO found 62.4%, 82.2%, and 93.6% of the plant-universal orthologs (**Supplementary Table S3**). In the *M.* *truncatula* genome, BUSCO found 95.5% of the plant-universal genes (**Supplementary Table S3**). The number of ECs found for each biosynthetic pathway based on KEGG-KAAS server searches overall showed similar results across the three transcriptomes and the genome: of the 410 separate pathways listed in KEGG, only 15 of them differed in their enzyme counts by more than 2 between any transcriptome or genome (**Table 1** and **Supplementary Table S4**). Transcripts coding for 11 out of the 14 enzymes in the sequiterpenoid/triterpenoid pathway were expressed in *M.* *truncatula* while only five and four enzymes were found in *C.* *thyrsiflorus* and *D.* *glomerata* transcriptomes, respectively; for the isoflavonoid biosynthesis pathway, 9 of the 14 enzymes were expressed in *M.* *truncatula*, while only one each was expressed in *C.* *thyrsiflorus* and *D.* *glomerata*.

**Table 1:** Enzymes in selected KEGG biosynthetic pathways represented in transcriptomes and *M.* *truncatula* genome, showing more than two enzyme differences per pathway among the three hosts.

KEGG pathway ID	KEGG pathway name	Total enzymes in KEGG pathway	*C. thyrsiflorus* (T)	*D. glomerata* (T)	*M. truncatula* (T)	*M. truncatula* (G)
130	Ubiquinone and other terpenoid-quinone biosynthesis	47	19	23	24	24
230	Purine metabolism	106	57	61	62	62
240	Pyrimidine metabolism	75	36	41	40	41
540	Lipopolysaccharide biosynthesis	33	8	12	13	13
670	One carbon pool by folate	24	9	13	13	13
710	Carbon fixation in photosynthetic organisms	27	19	21	23	23
730	Thiamine metabolism	29	14	16	18	18
790	Folate biosynthesis	30	11	14	15	15
860	Porphyrin and chlorophyll metabolism	87	23	30	32	32
906	Carotenoid biosynthesis	40	22	25	25	25
909	Sesquiterpenoid and triterpenoid biosynthesis	77	5	4	11	11
941	Flavonoid biosynthesis	19	16	13	13	15
943	Isoflavonoid biosynthesis	14	1	1	9	9
1200	Carbon metabolism	221	92	93	96	97
1210	2-Oxocarboxylic acid metabolism	58	30	29	32	32

*T, transcriptome; G, genome*.

### Annotation, Differential Gene Expression

The root + nodule transcriptomes of *C.* *thyrsiflorus* and *D.* *glomerata* and the genome of *M.* *truncatula* were annotated (by KO, EC, and/or GO) for 88.1%, 85.0%, and 60.9% of the genes, respectively (**Supplementary Table S5**). No abnormalities in the mean variance trend of gene expressions were found by limma (Ritchie et al., 2015), and there was a clear distinction in gene expression patterns between the roots and the nodules for all three species (**Supplementary Image S1**). Differential gene expression analysis found 19.2% (2,932 root-enhanced and 745 nodule-enhanced), 34.2% (2,726 root-enhanced and 2,550 nodule-enhanced), and 34.6% (4,819 root-enhanced and 4,965 nodule-enhanced) of genes significantly differentially expressed in the *C.* *thyrsiflorus, D.* *glomerata*, and *M.* *truncatula* transcriptomes, respectively.

In the GO enrichment analysis, although particular annotated functions (GO terms) were enriched in roots or nodules of an individual species, when the results were compared across the three species, no common or similar patterns of enrichment were found (**Supplementary Table S6**).

### Validation of RNA-seq With qPCR of cDNA Library

A subset of the gene expression patterns calculated based on RNA-seq were validated by qPCR. Of the 14 genes tested (seven genes in each of the two actinorhizal species), 13 showed similar log-fold changes in expression as the RNA-seq results. Of these 13 genes, eight of which were <2 log2-fold change apart from the RNA-seq results. *NRT1.8* in *C.* *thyrsiflorus* showed an expression pattern in conflict with the RNA-seq results (**Supplementary Table S7**).

### Orthology Predictions

Orthologs for each gene in the *C.* *thyrsiflorus* and *D.* *glomerata* transcriptomes and the *M.* *truncatula* genome were predicted independently using OrthoReD (Battenberg et al., 2017), a tree-based orthology prediction tool. OrthoReD predicted orthologs for all genes within the *C.* *thyrsiflorus* transcriptome, *D.* *glomerata* transcriptome, and the *M.* *truncatula* genome (total of 81,587 genes). Because OrthoReD predicts a set of orthologs for each gene independently, the predicted sets of orthologs for different genes are not always mutually exclusive. Thus, in order to avoid analyzing the same gene multiple times for the same analysis, orthologous sets were merged into 27,367 mutually exclusive groups (MergedOrthoGroups).

We note here that these orthologs are predicted based on the specific dataset that was analyzed. Including a more complete dataset for example by replacing the *C.* *thyrsiflorus* and *D.* *glomerata* transcriptomes with genomes, or by including more species may result in different predictions.

### Presence and Expression Patterns of RNS Pathway Genes

**FIGURE 1:**
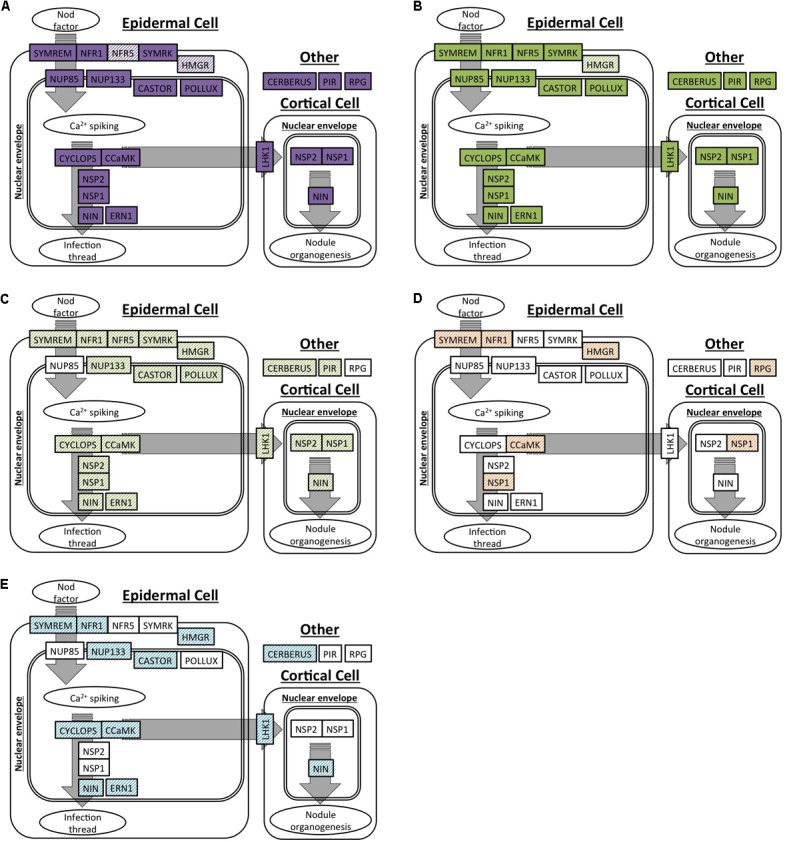
Orthologs or homologs of genes involved in the initiation of root nodule symbiosis identified in four actinorhizal hosts. A spatial and sequential model of genes (indicated as boxes) involved in the initiation of root nodule symbiosis based on *M.* *truncatula* and/or *L.* *japonicus*. Genes are diagrammed at locations where the model expects the gene product to be localized in the root tissue. Orthologs are shown as solid colors; homologs are shaded. **(A,B)** Genes with orthologs detected in this study: **(A)** *C.* *thyrsiflorus*, and **(B)** *D.* *glomerata*. **(C–E)** Respectively: homologs detected in *D.* *glomerata* according to Demina et al. (2013); homologs detected in *A.* *glutinosa* according to Hocher et al. (2011); homologs detected in *C.* *glauca* according to Hocher et al. (2011).

We selected 19 genes that are required for the development of nodules in *M. truncatula* and *L.* *japonicus* to specifically search for orthologs in *C.* *thyrsiflorus* and *D.* *glomerata* transcriptomes. Of these, OrthoReD identified orthologs for 17 and 18 genes in *C.* *thyrsiflorus* and *D.* *glomerata* transcriptomes, respectively (**Table 2**). These orthologs spanned from the most upstream step (e.g., *NFR1, SYMRK*) to *NIN*, one of the key transcription factors for RNS (**Figure 1**). OrthoReD found transcripts with high sequence similarity (putative homologs) at its intermediate step in orthology prediction for all 19 genes including *FNR5* and *HMGR* in *C.* *thyrsiflorus* and *HMGR* in *D.* *glomerata* (**Table 2**).

**Table 2:** Fold changes (nodule over root) and adjusted *p*-values of symbiotic pathway genes.

Gene Name	MergedOrtho Group	*C. thyrsiflorus* ortholog			*D. glomerata* ortholog			*M.* *truncatula*/*L.* *japonicus* ortholog			
		Gene ID	Log_2_FC	*P*-value	Gene ID	Log_2_FC	*P*-value	Gene ID	Log_2_FC	*P*-value	GenBank ID
NFR1	–	Ct_CtTrNR03429	-0.37	0.151	Dg_DgTrNR03578	2.75	0.000	Mt_Medtr5g 086130	-2.10	0.000	AY372406.1
					Dg_DgTrNR03579	-1.39	0.000				
					Dg_DgTrNR03580	2.54	0.000				
NFR5	–	Ct_CtTrNR01660, Ct_CtTrNR02016, Ct_CtTrNR02556, Ct_CtTrNR03429, Ct_CtTrNR03658, Ct_CtTrNR10088, Ct_CtTrNR13226, Ct_CtTrNR13284, Ct_CtTrNR14238, Ct_CtTrNR14293	Dg_DgTrNR09631	-1.52	0.000	Mt_Medtr5g019040	-2.43	0.000	DQ496250.1
SYMRK	MergedOrthoGroup005636	Ct_CtTrNR07134	-0.19	0.413	Dg_DgTrNR12393	0.46	0.000	Mt_Medtr5g030920	0.60	0.002	AF491998.1
SYMREM	MergedOrthoGroup006520	Ct_CtTrNR08408	2.10	0.003	Dg_DgTrNR05672	5.65	0.000	Mt_Medtr5g010590	-0.22	0.775	–
								Mt_Medtr8g097320	9.88	0.000	JQ061257.1
HMGR	–	Ct_CtTrNR06569, Ct_CtTrNR06801, Ct_CtTrNR08716, Ct_CtTrNR12229	Dg_DgTrNR07804, Dg_DgTrNR14611, Dg_DgTrNR14612	Mt_Medtr5g087550	-1.35	0.000	XM_003617018.2
NUP85	MergedOrthoGroup003212	Ct_CtTrNR03893	-0.87	0.020	Dg_DgTrNR02299	0.12	0.132	Mt_Medtr1g006690	-0.19	0.665	XM_003588338.2
NUP133	MergedOrthoGroup009404	Ct_CtTrNR12969	-0.20	0.263	Dg_DgTrNR15290	0.58	0.000	Lj_Lj2g3v3337540	–	–	AJ890251.1
								Lj_Lj0g3v0050449	–	–	
								Mt_Medtr5g097260	-0.48	0.064	–
CASTOR	MergedOrthoGroup008762	Ct_CtTrNR11902	-0.39	0.071	Dg_DgTrNR14656	0.37	0.001	Mt_Medtr7g117580	-0.23	0.431	FJ974130.1
POLLUX	MergedOrthoGroup009011	Ct_CtTrNR12292	-0.43	0.090	Dg_DgTrNR14917	-0.83	0.000	Mt_Medtr2g005870	1.61	0.000	AY497771.1
CCAMK	MergedOrthoGroup006145Sub001	Ct_CtTrNR07875	-0.49	0.064	Dg_DgTrNR14486	0.02	0.857	Mt_Medtr8g043970	0.59	0.004	AY496049.1
CYCLOPS	MergedOrthoGroup005459Sub002	Ct_CtTrNR09813	0.25	0.294	Dg_DgTrNR00459	1.60	0.000	Mt_Medtr5g026850	3.18	0.000	EF117279.1
NSP1	MergedOrthoGroup009317	Ct_CtTrNR12805	-0.10	0.595	Dg_DgTrNR11911	0.96	0.000	Mt_Medtr3g085310	0.00	1.000	–
								Mt_Medtr8g020840	1.62	0.000	AJ972478.1
NSP2	MergedOrthoGroup000090	Ct_CtTrNR00101	0.50	0.232	Dg_DgTrNR00363	-1.38	0.000	Mt_Medtr3g072710	-1.34	0.000	AJ832138.1
					Dg_DgTrNR01580	0.99	0.001	Mt_Medtr5g058860	-2.59	0.000	–
ERN1	MergedOrthoGroup010414	Ct_CtTrNR15219	-2.56	0.003	Dg_DgTrNR02845	-0.66	0.006	Mt_Medtr6g029180	-2.53	0.000	–
					Dg_DgTrNR10008	4.95	0.000	Mt_Medtr7g085810	-0.39	0.373	EU038802.2
NIN	MergedOrthoGroup008966	Ct_CtTrNR12221	5.48	0.003	Dg_DgTrNR00885	8.30	0.000	Mt_Medtr5g099060	7.42	0.000	FJ719774.1
		Ct_CtTrNR12222	6.47	0.004	Dg_DgTrNR00886	7.56	0.000				
LHK1	MergedOrthoGroup007561	Ct_CtTrNR09972	1.16	0.156	Dg_DgTrNR03717	-0.92	0.000	Lj_Lj0g3v0108729	–	–	DQ848999.1
								Mt_Medtr2g067240	0.00	1.000	–
								Mt_Medtr5g044100	0.00	1.000	–
								Mt_Medtr8g106150	-0.22	0.524	–
PIR	–	Ct_CtTrNR13403	0.13	0.688	Dg_DgTrNR01709	0.17	0.048	Lj_Lj1g3v5020900	–	–	AM946364.1
					Dg_DgTrNR07598	-0.51	0.000				
RPG	MergedOrthoGroup006667Sub001	Ct_CtTrNR08628	-0.78	0.022	Dg_DgTrNR05600	8.90	0.000	Mt_Medtr1g090807	6.45	0.000	DQ854741.1
		Ct_CtTrNR14741	5.14	0.003	Dg_DgTrNR07056	-0.25	0.102				
CERBERUS	MergedOrthoGroup002490Sub002	Ct_CtTrNR11435	0.24	0.265	Dg_DgTrNR05473	0.87	0.000	Mt_Medtr1g090320	1.25	0.000	EU926661.1


*For each symbiotic pathway gene, the corresponding MergedOrthoGroup and the ortholog(s) are shown. Each ortholog also has its log2-fold change and adjusted *p*-values are indicated. The yellow cells are not orthologs, but are putative homologs found by OrthoReD. All genes included in this table were selected from Kouchi et al. (2010) and/or Oldroyd (2013)*.

Three of the 19 RNS pathway genes were found to be universally nodule-enhanced: *SYMREM* (Lefebvre et al., 2010), a remorin that is known to interact with *NFR1*, in the most upstream portion of the RNS pathway; *NIN* (Schauser et al., 1999), a key transcription factor that is one of the more downstream RNS pathway genes required for nodule organogenesis; and *RPG* (Arrighi et al., 2008), a gene required for the proper growth and regulation of the infection thread that is key to the establishment of RNS in *M.* *truncatula* (**Table 2**). Fourteen of the RNS pathways genes were found to have an ortholog in all three taxa, but there was not a significant universal enhancement of gene expression in this group of genes (i.e., passing both criteria for fold change and *p*-value) in a single tissue (root vs. nodule). At the same time, none of these genes showed a significant conflicting pattern of gene expression (nodule-enhanced in one ortholog while root-enhanced in another) across different species (**Table 2**).

### Expression Similarity Analysis

**FIGURE 2:**
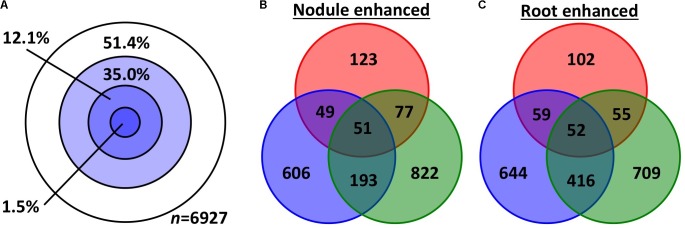
Gene expression overlap among the representative MergedOrthoGroups. **(A)** Concentric circles each represent MergedOrthoGroups that showed significant enhancement in different number of species: The innermost circle represents the MergedOrthoGroups showing enhancement in all three species; the next circle represents significant enhancement in two species then one, and the outermost while circle is for MergedOrthoGroups showing no significant enhancement in any species. **(B,C)** The number of MergedOrthoGroups that showed significant enhancement in nodules **(B)** or in roots **(C)**. The three colors red, blue, and green correspond to *C.* *thyrsiflorus, D.* *glomerata*, and *M.* *truncatula*, respectively.

We used two approaches to compare differential (root vs. nodule) gene expression patterns among the three species, on a pairwise basis. We parsed out 3,894 whole and 3,033 subsets of MergedOrthoGroups (total of 6,927 MergedOrthoGroups) as the representative MergedOrthoGroups for these analyses (**Supplementary Table S8**). Of the 19 RNS pathway genes, 15 were represented within these MergedOrthoGroups (**Table 2**). The representative MergedOrthoGroups included 49.2%, 51.0%, and 38.7% of the root + nodule transcriptomes of *C.* *thyrsiflorus, D.* *glomerata*, and *M.* *truncatula*, respectively. Among the 6,927 representative MergedOrthoGroups, 3,365 (48.6%) of them were enhanced in at least one tissue in at least one of the three species (**Figure 2**). Among these, there were 103 core MergedOrthoGroups that showed universal enhancement either in the nodule (*n* = 51) or in the root (*n* = 52) across the three species (**Figure 2**). The nodule-enhanced core MergedOrthoGroup included sets of orthologs for *SYMREM, NIN*, and *RPG* as described above in section “*Presence of RNS Pathway Genes and Their Gene Expression Patterns*.”

**FIGURE 3:**
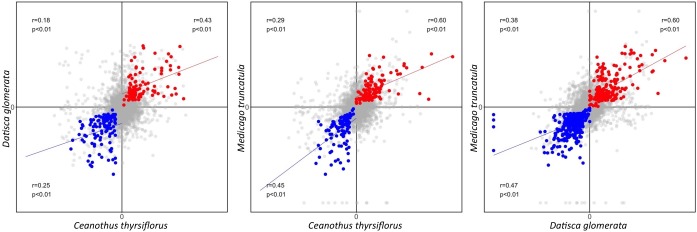
Pearson correlation of gene expression fold change between two species. Gene expression fold change (nodule over root) is plotted as a comparison between each pair of species. The Pearson correlation coefficient calculated from all representative MergedOrthoGroup (in gray) is displayed in the second quadrant along with the corresponding *p*-value. The Pearson correlation coefficient of root co-enhanced MergedOrthoGroups (in blue) and nodule co-enhanced MergedOrthoGroups (in red) are displayed in the third and the first quadrant, respectively. Blue and the red lines each show the regression lines of root and nodule co-enhanced MergedOrthoGroups, respectively.

First, based on the Pearson correlation coefficient, we found that the gene expression fold changes between roots and nodules were weakly to moderately correlated (*r* = 0.18–0.38 for the three pairwise species comparisons) when all 6,927 representative MergedOrthoGroups were analyzed. The correlations were stronger for MergedOrthoGroups whose expression was enhanced in roots compared to nodules, of both species being compared (*r* = 0.25–0.47), and strongest for MergedOrthoGroups whose expression was enhanced in the nodules of both species being compared (*r* = 0.43–0.60) (**Figure 3**). The correlations were statistically significant in all cases (*p* < 0.01) (**Figure 3**).

Second, the degree and significance of overall similarity in gene expression between each pair of species was assessed using a scale, which we call the dissonance score (see section “*Expression Similarity Analysis”* in Materials and Methods for detail and **Supplementary Image S2** for a summary). Dissonance scores across all MergedOrthoGroups for all pairwise species comparison showed that the expression patterns of nodules compared to roots in the three species were more similar to each other than by random chance in all pairwise comparisons across the three species. In fact the dissonance score from any of the 10,000 random permutations conducted for each pairwise comparison was never lower than the observed value (*p* < 0.0001).

### Gene Ontology (GO) Enrichment Analysis of the Core MergedOrthoGroups

Gene Ontology terms (GO terms) provide gene annotations that permit grouping into integrated biological processes (Ashburner et al., 2000; Gene Ontology Consortium, 2017). A few GO terms were universally enriched in the comparison of core MergedOrthoGroups across the three host species: nitrate transport (GO:0015706), and metabolic processes of zeatin and trans-zeatin (GO:0033397, GO:0033398, GO:0033400, GO:0033466) were universally enriched in nodules; while strigolactone biosynthesis (GO:1901600, GO:1901601), secondary shoot formation (GO:0010223, GO:0010346), and cellular response to NO (GO:0034614, GO:0071731, GO:0071732, and GO:1902170) were universally enriched in roots (**Table 3** and **Supplementary Image S3**).

**Table 3:** Enriched Gene Ontology (GO) terms among the root- and the nodule-enhanced core MergedOrthoGroups that are shared across the three species.

GO ID	GO term	*P*-value (*C.* *thyrsiflorus*)	*P*-value (*D.* *glomerata*)	*P*-value (*M.* *truncatula*)
**(A) Nodule-enhanced GO terms shared across the three species.**		
GO:0009690	Cytokinin metabolic process	4.9.E-05	2.5.E-04	4.2.E-04
GO:0015706	Nitrate transport	5.8.E-05	2.3.E-03	3.0.E-03
GO:0034754	Cellular hormone metabolic process	2.0.E-04	4.4.E-04	1.1.E-03
GO:0015698	Inorganic anion transport	4.0.E-04	2.9.E-03	5.4.E-03
GO:0006820	Anion transport	4.2.E-04	5.9.E-04	2.1.E-04
GO:0042445	Hormone metabolic process	8.1.E-04	3.8.E-04	1.1.E-03
GO:0010817	Regulation of hormone levels	3.4.E-03	2.8.E-03	4.5.E-03
GO:0010028	Xanthophyll cycle	3.8.E-03	1.8.E-05	1.6.E-05
GO:0015882	L-Ascorbic acid transport	3.8.E-03	1.8.E-05	1.6.E-05
GO:0033397	Zeatin metabolic process	3.8.E-03	1.8.E-05	1.6.E-05
GO:0033398	Zeatin biosynthetic process	3.8.E-03	1.8.E-05	1.6.E-05
GO:0033400	*Trans*-zeatin metabolic process	3.8.E-03	1.8.E-05	1.6.E-05
GO:0033466	*Trans*-zeatin biosynthetic process	3.8.E-03	1.8.E-05	1.6.E-05
GO:0010101	Post-embryonic root morphogenesis	6.7.E-03	9.2.E-04	1.3.E-03
GO:0010102	Lateral root morphogenesis	0.00665	0.00092	0.00132
GO:0051180	Vitamin transport	0.00767	0.000054	0.00019
**(B) Root-enhanced GO terms shared across the three species.**		
GO:1901334	Lactone metabolic process	1.4.E-06	4.3.E-07	5.7.E-11
GO:1901336	Lactone biosynthetic process	1.4.E-06	4.3.E-07	5.7.E-11
GO:1901600	Strigolactone metabolic process	1.4.E-06	4.3.E-07	5.7.E-11
GO:1901601	Strigolactone biosynthetic process	1.4.E-06	4.3.E-07	5.7.E-11
GO:0034756	Regulation of iron ion transport	2.8.E-06	3.4.E-04	1.4.E-04
GO:0071732	Cellular response to nitric oxide	2.8.E-06	5.9.E-06	1.4.E-09
GO:1902170	Cellular response to reactive nitrogen species	2.8.E-06	1.3.E-05	5.8.E-09
GO:0071281	Cellular response to iron ion	3.9.E-06	4.5.E-03	2.5.E-04
GO:0071731	Response to nitric oxide	4.8.E-06	8.8.E-06	5.8.E-09
GO:0010039	Response to iron ion	9.6.E-06	6.4.E-03	4.3.E-04
GO:0010223	Secondary shoot formation	6.1.E-05	3.7.E-05	2.6.E-08
GO:0010346	Shoot axis formation	6.1.E-05	3.7.E-05	2.6.E-08
GO:0071241	Cellular response to inorganic substance	6.1.E-05	1.2.E-03	4.9.E-07
GO:0001763	Morphogenesis of a branching structure	1.1.E-04	6.9.E-05	3.7.E-08
GO:0010959	Regulation of metal ion transport	1.5.E-04	2.3.E-03	2.8.E-03
GO:0042445	Hormone metabolic process	2.7.E-04	4.2.E-05	3.1.E-03
GO:0034614	Cellular response to reactive oxygen species	9.5.E-04	4.4.E-04	3.6.E-07
GO:0016106	Sesquiterpenoid biosynthetic process	1.9.E-03	3.7.E-05	2.1.E-08
GO:0006714	Sesquiterpenoid metabolic process	0.00242	0.000069	0.00000012
GO:1905393	Plant organ formation	0.00271	0.00484	0.000056
GO:0006812	Cation transport	0.00378	0.00605	0.00175
GO:0055114	Oxidation–reduction process	0.00409	0.00986	0.00451
GO:0071369	Cellular response to ethylene stimulus	0.00433	0.00404	0.00015
GO:0016114	Terpenoid biosynthetic process	0.00457	0.00056	0.0000089
GO:0034599	Cellular response to oxidative stress	0.0057	0.0026	0.0000052
GO:0055085	Transmembrane transport	0.00588	0.0012	0.00216
GO:0006811	Ion transport	0.00659	0.00782	0.00047
GO:0006721	Terpenoid metabolic process	0.00659	0.00099	0.000017

Enrichment of GO terms related to a nitrate transporter in the nodule was due to universal up-regulation of a set of orthologs (MergedOrthoGroup008002) corresponding to *NRT1.8* in *Arabidopsis*, a low-affinity nitrate transporter hypothesized to export nitrate from xylem to xylem parenchyma cell (Li et al., 2010).

The set of orthologs responsible for the enrichment of GO terms related to cytokinin (MergedOrthoGroup005893) was *CYP735A*, a cytokinin hydroxylase that catalyzes the biosynthesis of trans-zeatin in *Arabidopsis* (Takei et al., 2004). We also found another set of orthologs (MergedOrthoGroup008876) coding for IPT (isopentenyl transferase) (Azarakhsh et al., 2015) up-regulated in the nodules of *C.* *thyrsiflorus* and *D.* *glomerata*, but down-regulated in the *M.* *truncatula* nodule.

Enrichment of GO terms related to strigolactone biosynthesis in the roots was due to universal up-regulation of two sets of orthologs: *MAX4* (MergedOrthoGroup000413), a likely carotenoid dioxygenase (Sorefan et al., 2003), *D27* (MergedOrthoGroup006746), a carotenoid isomerase (van Zeijl et al., 2015a). A third set of universally up-regulated orthologs, *DLK2* (MergedOrthoGroup000413), has been classified under the strigolactone GO term, but was recently shown not to be involved in strigolactone signaling (Waters et al., 2012; Bennett et al., 2016; Vegh et al., 2017). These three sets of orthologs were also annotated to be related to secondary shoot formation.

Enrichment of the GO term related to NO in the roots was due to universal up-regulation of two sets of orthologs: MergedOrthoGroup006609, a gene coding for a histidine kinase (*AHK5*) (Iwama et al., 2007), and MergedOrthoGroup001248Sub001 which did not have a well-documented member.

### *dN/dS* Analysis

We used a molecular phylogenetic approach to model changes in selection pressure along key branches in the phylogenies of selected MergedOrthoGroups. These consisted of 3,894 MergedOrthoGroups that (1) contained at least one member from each of *C.* *thyrsiflorus, D.* *glomerata*, and *M.* *truncatula*, and (2) were identical to a set of orthologs predicted from a single gene with respect to all 15 species, were used in this analysis. These included 58 core genes (showing a consistent pattern of differential expression among the three target nitrogen-fixing species) as well as 9 of the 19 known RNS pathway genes: *SYMRK, NUP85, NUP133, CASTOR, POLLUX, NSP2, ERN1, NIN*, and *LHK1*.

**FIGURE 4:**
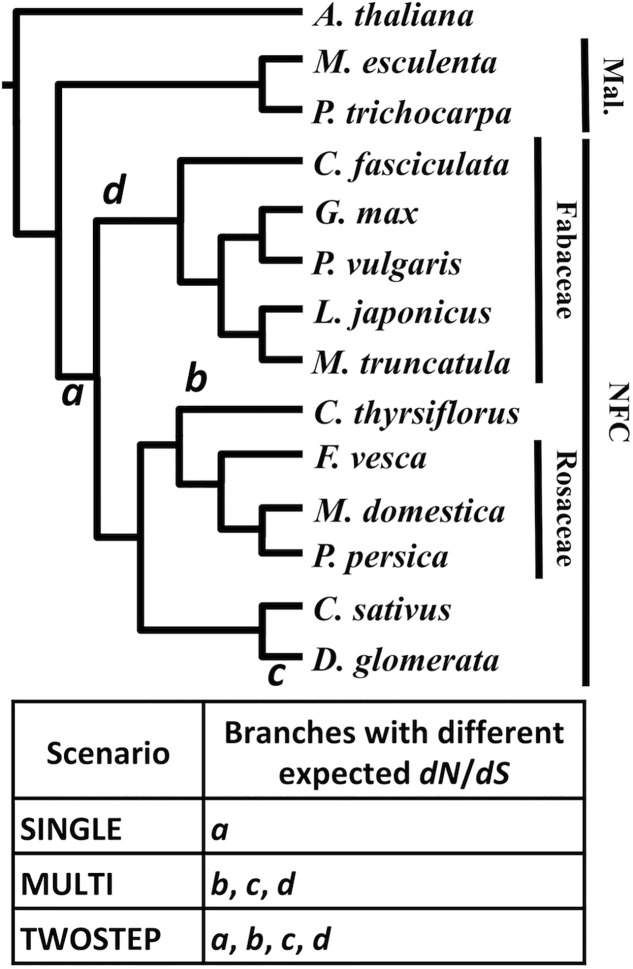
Different scenarios tested based on competing hypotheses of the origin of RNS. A phylogenetic tree of the NFC and its outgroup. The branches *a* through *d* designate the base of the NFC (*a*), *C.* *thyrsiflorus* (*b*), *D.* *glomerata* (*c*), and the legumes (*d*), respectively. The table below indicates the three hypothesis-based scenarios and where each scenario expects a change in *dN*/*dS* in the phylogeny. Species without a family label each belong to a separate family: *A.* *thaliana* (Brassicaceae), *M*. *esculenta* (Euphorbiaceae), *P.* *trichocarpa* (Salicaceae), *C.* *thyrsiflorus* (Rhamnaceae), *C.* *sativus* (Cucurbitaceae), and *D.* *glomerata* (Datiscaceae). Mal., Malpighiales. The topology of the tree is based on Angiosperm Phylogeny Group III (APG, 2009).

For each MergedOrthoGroup, the likelihoods of up to three different hypothesis-based scenarios (SINGLE, MULTI, and TWOSTEP), each of which predicts a different timing for a change in *dN*/*dS*, were compared to a NULL scenario, which assumes a constant *dN*/*dS* throughout the gene phylogeny (**Figure 4**).

For each of the 3,894 MergedOrthoGroups, we tested whether any of the three hypothesis-based scenario (MULTI, SINGLE, or TWOSTEP) was significantly better than the NULL scenario (see section “*dN/dS* Analysis” in Materials and Methods for detail and **Figure 4** for a summary). Among the 3,894 MergedOrthoGroups, 2,668 did not reject the NULL scenario and the remaining 1,226 did not reject one or more of the alternative scenarios (MULTI, SINGLE, or TWOSTEP).

Focusing on the branch at the base of the NFC (branch *a* in **Figure 4**), 1,166 MergedOrthoGroups did not reject scenarios that predict a change in selection pressure here (SINGLE and/or TWOSTEP). Of these, 364 MergedOrthoGroups (31.2%) rejected neither SINGLE nor TWOSTEP, 6 (0.5%) rejected TWOSTEP, and 796 (68.3%) rejected SINGLE. For branches leading to *C.* *thyrsiflorus, D.* *glomerata*, and the legumes (branches *b, c, d* in **Figure 4**, respectively), 1,220 MergedOrthoGroups supported scenarios that predict changes in selection pressure along those branches (MULTI and/or TWOSTEP). Of these, 1,150 MergedOrthoGroups (94.3%) rejected neither MULTI nor TWOSTEP, 60 (4.9%) rejected TWOSTEP, and 10 (0.8%) rejected MULTI (**Table 4**).

**Table 4:** Number and fractions of MergedOrthoGroups that show a change in *dN*/*dS* at branches *a, b, c*, and *d* in **Figure 4**.

All MergedOrthoGroups			Reject TWOSTEP	Reject neither	Reject SINGLE	Total
	*a*	Count	6	364	796	1,166
		Percentage	0.5%	31.2%	68.3%	
			Reject TWOSTEP	Reject neither	Reject MULTI	Total
	*b, c, d*	Count	60	1150	10	1,220
		Percentage	4.9%	94.3%	0.8%	
Core MergedOrthoGroups			Reject TWOSTEP	Reject neither	Reject SINGLE	Total
	*a*	Count	0	4	13	17
		Percentage	0.0%	23.5%	76.5%	
			Reject TWOSTEP	Reject neither	Reject MULTI	Total
	*b, c, d*	Count	2	17	0	19
		Percentage	10.5%	89.5%	0.0%	

The 1,226 MergedOrthoGroups included a subset of core MergedOrthoGroups (9 universally nodule-enhanced, 10 universally root-enhanced). The pattern of scenarios not rejected in the analysis of the core MergedOrthoGroups was similar to that of the overall results: Among the core MergedOrthoGroups that did not reject a change of selection pressure at branch *a* (i.e., either SINGLE or TWOSTEP), a majority (76.5%) rejected the SINGLE scenario. In addition, among the core MergedOrthoGroups that did not reject changes in selection pressure at branches *b, c*, and *d*, most (89.5%) rejected neither the MULTI nor the TWOSTEP scenario (**Table 4**). Among the RNS pathway genes, only *ERN1* rejected the NULL scenario and supported MULTI and TWOSTEP scenarios.

## Discussion

### Newly Assembled Transcriptomes Are of High Quality

Both *C.* *thyrsiflorus* and *D.* *glomerata* root + nodule transcriptomes scored well in multiple measures of quality (e.g., proper insert size, long N50, and high % fragment mapped) throughout the assembly process. After the assembly, KEGG annotation found the two transcriptomes to have similar numbers of ECs to the *M.* *truncatula* transcriptome (and even to the *M.* *truncatula* genome) for most of the pathways (**Table 1** and **Supplementary Table S4**). Both transcriptomes were annotated (by KO, EC, and/or GO) for >85% of the transcripts. Moreover, BUSCO found 62.4%, 82.2% of the plant-universal orthologs for *C.* *thyrsiflorus* and *D.* *glomerata*, respectively. Furthermore, all the qPCR validated the RNA-seq results except for one gene (**Supplementary Table S7**). This all together indicate that the root + nodule transcriptomes of *C.* *thyrsiflorus* and *D.* *glomerata* are of high quality.

### Nodule Gene Expression Patterns in *C. thyrsiflorus, D. glomerata*, and *M. truncatula* Are More Similar to Each Other Than Would be Expected by Random Chance

Our analysis supports the homology (shared by common descent) of RNS among the three plant species based on multiple lines of evidence. First, orthologs for most of the 19 RNS pathway genes required for the proper nodulation in *M.* *truncatula* were present and expressed in the nodules of *C.* *thyrsiflorus* (17 out of 19 found) and *D.* *glomerata* (18 out of 19 found) (**Figure 1**). Transcripts with high sequence similarity were found, even for the few genes that did not have an ortholog predicted (**Figure 1** and **Table 2**). Results of this orthology-based analysis strengthens previous homology-based reports (inferred based on high scores in BLAST searches) throughout the RNS pathway in *D.* *glomerata, Alnus* *glutinosa* (Betulaceae, Fagales) and *Casuarina* *glauca* (Casuarinaceae, Fagales) (Hocher et al., 2011; Demina et al., 2013); and are consistent with reports demonstrating functional equivalents (presumed orthologs) of a specific member of the RNS pathway, such as *SYMRK* (Markmann et al., 2008), *CCaMK* (Svistoonoff et al., 2013), and *NIN* (Clavijo et al., 2015), across multiple lineages. This is the first time, to our knowledge, that the entire pathway has been detected comprehensively in the context of a phylogenetically based orthology framework. It is also important to emphasize that the orthologs identified in our analysis included *NFR1, NSP1, ERN1*, and *NIN*, genes not shared with the more ancient AM symbiosis (Oldroyd, 2013). The presence and the expression of orthologs across these three species indicates that their existence predates the NFC, which is required for the RNS to share a common function and common origin across them.

In all pairwise comparisons of gene expression patterns between two species, we found a moderate to strong positive correlation for genes that were significantly enhanced in the nodules (*r* = 0.43–0.60); and there was a weak to moderate positive correlation for genes that were significantly enhanced in the roots (*r* = 0.25–0.47). The correlation was much weaker when all genes (including genes that are not necessarily presumed to be involved in RNS) were compared at once across two transcriptomes (*r* = 0.18–0.38) (**Figure 2**). While it is common to describe Pearson correlation coefficients of *r* = 0.20–0.39, *r* = 0.40–0.59, *r* = 0.6–0.79, as indicating weak, moderate, and strong correlation, respectively, meaningful interpretations of particular values depend on the context in which they were obtained, in this case, comparisons of transcriptomes across a considerable phylogenetic distance. For example, the Pearson correlation coefficient between the gene expression patterns between two sets of 20 plants in one species, *Arabidopsis* *thaliana*, was 0.81 (Kempema et al., 2007). By contrast, the comparisons made in our study are between pairs of different plant species that have been diverging for nearly 100 million years (Bell et al., 2010); thus, we consider that the values of *r* = 0.43–0.60 found for nodule-enhanced genes indicate a high degree of similarity and conservation, compared with 0.18–0.38 for all genes.

Finally, permutation tests based on the dissonance scores indicated that the overall gene expression patterns of the nodules in the three RNS hosts tested are more similar to each other than expected by random chance (*p* < 0.0001).

These results strongly support the homology of RNS in all three lineages, i.e., that their similarity is due to common descent. It is possible that other factors, such as similarity in the age of these tissues across the three transcriptomes, may have contributed to the similarity of the gene expression patterns. An increased spatiotemporal resolution for *C.* *thyrsiflorus* and *D.* *glomerata*, as obtained for *M. truncatula* through a time course transcriptome (Larrainzar et al., 2015) or tissue-specific transcriptome (Roux et al., 2014) would provide further clarity.

### Features of RNS Conserved Among the Three Lineages

Orthologs of 17 or 18 of the 19 RNS pathway genes were expressed in the root + nodule transcriptomes of *C.* *thyrsiflorus* and *D.* *glomerata*, respectively, and three of them (*SYMREM, NIN*, and *RPG*) were universally nodule-enhanced (**Table 2**). The up-regulation of *SYMREM, NIN*, and RPG in the nodules was also found in *A.* *glutinosa*, and *C.* *glauca* (Hocher et al., 2011), and is consistent with what was found for *NIN* in *D.* *glomerata* (Demina et al., 2013). With the inclusion of *C.* *thyrsiflorus*, we now show that an up-regulation of *SYMREM, NIN*, and *RPG* in the nodules of RNS hosts is found in all four orders within the NFC.

Because the initiation and establishment of RNS consists of multiple developmental stages (Pawlowski and Demchenko, 2012; Roux et al., 2014), a high degree of spatiotemporal resolution is crucial to accurately trace the expression pattern of a gene (Roux et al., 2014; Larrainzar et al., 2015): Genes up-regulated only at a specific stage of nodule development may not be up-regulated within a transcriptome that is inclusive of the entire nodule. Thus, genes that are up-regulated (relative to root) in the whole nodule are expected either (1) to be so strongly nodule-enhanced in a given stage that they are detected as up-regulated even after averaging expression values over the whole nodule, or (2) to be nodule-enhanced throughout the process of nodulation. The latter expression pattern has been well documented in the case of *NIN* (Schauser et al., 1999). For the remaining genes, whose orthologs were not universally up-regulated in either nodules or roots (some were up-regulated in two hosts and non-significant in the third), a higher resolution of space and time would be helpful for accurate comparisons across species.

Among the processes found to be universally enriched in either nodules or in roots among the core genes, we focused on the following four processes based on the potential relevance to RNS.

#### Nitrate Transporter

Orthologs of *NRT1.8*, a low-affinity nitrate transporter, were significantly up-regulated in the nodules of all three species of RNS hosts. In *A. thaliana, NRT1.8* is up-regulated by nitrate, and is hypothesized to export nitrate from xylem conducting cells to xylem parenchyma (Li et al., 2010). In *M.* *truncatula*, 50% of the expression of *NRT1.8* ortholog was located in Zone I (Roux et al., 2014), which corresponds to the nodule meristem. However, there are no functional conducting xylem elements in root meristems of higher plants, which would be the tissue equivalent of Zone I of the root nodule. Moreover, nitrate is known to suppress nodulation in *Ceanothus* (Thomas and Berry, 1989) and in *L.* *japonicus* (Soyano et al., 2014). In *L.* *japonicus*, nitrate and *NIN* work antagonistically against each other (Soyano et al., 2014): *NIN* expression suppresses the activation of nitrate-induced genes while nitrate suppresses the activation of *NF-YA1* and *NF-YB1*, the known targets of *NIN*. Taken together, these lines of evidence suggest that the function of *NRT1.8* in the root nodules is not related to nitrate. While the understanding of *NRT1.8* is still limited, the *NRT1* family proteins are also capable of transporting signaling compounds and phytohormones: In *A.* *thaliana, NRT1.1, NRT1.2*, and *NRT1.10* are involved in the transport of IAA, ABA, and glucosinolates, respectively (Chiba et al., 2015).

#### Strigolactone Biosynthesis

The genetic dissection of RNS has revealed that the pathway for its establishment shares many of its genes with the more ancient pathway to form AM symbiosis (Oldroyd, 2013). It is thus of substantial importance that *D27* and *MAX4*, genes coding for the enzymes of the first and the third steps of strigolactone biosynthesis (Ruyter-Spira et al., 2013) are apparently suppressed in the nodules of all three nitrogen-fixing hosts (**Table 3B**). Strigolactone has a number of functions in plants (Besserer et al., 2006; Gomez-Roldan et al., 2008), particularly as a key regulator in root development, with possible regulatory feedback interactions with auxin transport and metabolism (Kapulnik and Koltai, 2014). Strigolactone is also a signaling molecule that initiates AM symbiosis by stimulating the branching and growth of hyphae of the AM fungi (Besserer et al., 2006). Within the mature AM symbiotic tissue, however, strigolactone is down-regulated (Kapulnik and Koltai, 2014).

A similar pattern may be inferred for RNS. Strigolactone has been shown to play a role in promoting nodulation in *Pisum* *sativum*, in that a strigolactone mutant formed fewer nodules than wild-type (Foo and Davies, 2011), although, strigolactone is not required for nodulation, since nodulation does occur in the mutant. The influence of strigolactone on nodulation in *P. sativum* seems to be limited to the early stage of infection thread formation (McAdam et al., 2017).

Thus, the down-regulation of strigolactone biosynthesis in the nodule tissue of three phylogenetically distinct hosts that was observed in this study could be a function derived from AM symbiosis, to inhibit a portion of the root system from forming further infection sites, or to limit a stage of root development from further growth, allowing for nodule development or maintenance.

The enrichment of the GO term related to secondary shoot formation in the root transcriptome is likely related to the pattern of strigolactone gene expression observed, since *MAX4* plays a role in regulating branching in the shoot (Sorefan et al., 2003).

#### Cytokinin Biosynthesis

Up-regulation of genes associated with cytokinin biosynthesis and/or metabolism during the establishment of RNS is well documented in *L.* *japonicus* and *M.* *truncatula* (Tirichine et al., 2007; van Zeijl et al., 2015b; Gamas et al., 2017). Cytokinin in legume RNS is a major signal to the cortical cells to initiate nodule organogenesis by induction of *NIN* through the activation of a cytokinin receptor *LHK1* (Tirichine et al., 2007). Since the up-regulation of *NIN* has been found in the nodules of *A.* *glutinosa, C.* *glauca*, and *D.* *glomerata* (Hocher et al., 2011; Demina et al., 2013), *NIN* is considered to play a major role in RNS in actinorhizal plants as well as in the legumes. We found a universal up-regulation of *CYP735A*, a gene coding for the biosynthesis of trans-zeatin. *IPT* was also up-regulated in the nodules in *C.* *thyrsiflorus* and *D.* *glomerata*. Although *IPT* was down-regulated overall in *M.* *truncatula*, this is likely explained by the fact that >98% of *M.* *truncatula* *IPT* ortholog (Medtr2g022140) expression was restricted to the pre-infected cells (Roux et al., 2014).

#### Nitric Oxide Response

The cellular response to NO was universally down-regulated in the nodules of the three host plants in this study. Two sets of orthologs responsible for this pattern were detected, but only one had an assigned name: *AHK5*, a histidine kinase originally identified in *A.* *thaliana* as a regulator of stress response in guard cells (Desikan et al., 2008). In *M.* *truncatula*, NO was found to be a regulator of nodule senescence: increased and decreased levels of NO led to quickening and delay of nodule senescence, respectively (Cam et al., 2012). Since the nodules used in this study were all in relatively early stages of development, NO production in the nodules would not be expected to be high. Moreover, NO binds to and reduces activity of glutamine synthetase (Melo et al., 2011), the key enzyme in primary N assimilation. Thus, the down-regulation of *AHK5* would be important to maintain low concentrations of NO in the nodules. Alternatively, in *Arabidopsis, AHK5* is known to confer resistance to pathogens such as *Pseudomonas* *syringae* and *Botrytis* *cinerea* (Pham et al., 2012). Moreover, *AHK5* was most highly expressed in the roots of *A. thaliana* (Desikan et al., 2008). An inverse relationship exists between the host plant immune response and symbiotic processes established in the root nodules (Toth and Stacey, 2015). Down-regulation of *AHK5* could be part of the mechanism that enables the harboring of bacterial cells within the plant cells.

### *dN/dS* Analysis Disfavored the Single-Origin Hypothesis

Of the 3,894 MergedOrthoGroups, 31.5% (1,226) rejected the NULL scenario, which assumes a single rate of *dN*/*dS* throughout the tree. Of these 1,226 MergedOrthoGroups, 95.1% (1,166) supported a change in selection pressure at the base of the NFC. Among these, only 0.5% (6) rejected the TWOSTEP scenario while 68.3% (796) rejected the SINGLE scenario. The 58 core genes (MergedOrthoGroups), which should be strong candidates for playing key roles in the evolutionary origin of RNS, showed the same general pattern: 32.8% (19) rejected the NULL scenario, of which 89.5% (17) supported a change in selection pressure at the base of the NFC. Among these, 76.5% (13) rejected the SINGLE scenario, but none rejected the TWOSTEP scenario. We did not determine how many, if any, of the MergedOrthoGroups (i.e., sets of orthologous genes) are in fact the gene(s) that gave rise to RNS; thus our findings do not reject the single-origin hypothesis. However, the results of analyzing nearly 4,000 genes clearly disfavor the single-origin hypothesis.

Even the MergedOrthoGroups that rejected the TWOSTEP scenario are not in conflict with the two-step hypothesis, because the two-step hypothesis does not require the same gene to be responsible for both the gain-of-predisposition and the subsequent gain-of-function.

## Conclusion

The evolution of RNS represents a major event in the biology of plant-microbe interactions (Doyle, 2016), and different explanations of the evolutionary origins have been proposed. We have demonstrated the genetic homology of RNS in the three lineages based on the presence of RNS pathway orthologs and the high similarity of gene expression patterns across the three species, thus demonstrating that RNS shares a common evolutionary event at the base of the NFC. At the same time, we show that most genes (regardless of whether the gene is involved in the process of RNS or not) that experience change in selection pressure at the base of the NFC also experienced subsequent changes in selection pressure at the base of each RNS host lineage. Taken together, our results are most consistent with the two-step hypothesis of the origin of RNS. The work of Werner et al. (2014) supported the two-step hypothesis, but was based on a single trait (capability to establish RNS) and had been criticized for being based on a flawed phylogenetic tree (Doyle, 2016; LPWG, 2017). Our findings provide additional support for the two-step hypothesis.

On the other hand, two recent papers suggest a more ancient origin of functional RNS within the NFC followed by multiple losses (Griesmann et al., 2018; van Velzen et al., 2018). In Cannabaceae (Rosales), *Parasponia* retains the capability for RNS, whereas closely related *Trema* has lost it (van Velzen et al., 2018). Within a single genus, *Dryas* *octopetala* (Rosaceae) apparently does not form root nodules, whereas other *Dryas* species retain this trait (Becking, 1984). In a larger scale, two recent studies found that *NIN* and *RPG* have been lost among plants within the NFC that are not RNS hosts multiple times (Griesmann et al., 2018; van Velzen et al., 2018). Since the known functions of these genes are specific to RNS, multiple losses are difficult to explain under two-step hypothesis where these genes would be maintained for millions of years after the gain-of-predisposition event until the gain-of-function event.

Based on the known phylogenetic distribution of RNS hosts, the gain-of-predisposition at the MRCA of the NFC followed by a gain-of-function has been postulated as a parsimonious hypothesis since the discovery of the NFC (Soltis et al., 1995). What is the genetic nature of the predisposition assumed in the two-step hypothesis? Natural selection can only operate on a “predisposition” if the predisposition has a function of its own. Otherwise, the propensity for a gain-of-function could not have been conserved for tens of millions of years in multiple lineages (Doyle, 2011; Werner et al., 2014; Li et al., 2015). Likewise, a single-origin hypothesis needs an explanation for its apparently unparsimonious distribution of RNS hosts within the NFC. The high cost of RNS might be an explanation (Griesmann et al., 2018), but no direct evidence is available yet. In either case, the key to answer this question depends on an understanding of the genetic underpinnings that led to RNS, which still remains incomplete.

## Materials and Methods

### Plant Growth Conditions

*Ceanothus* *thyrsiflorus* var. *thyrsiflorus* plants as rooted cuttings were purchased from Cornflower Farms (Elk Grove, CA, United States). Plants were grown in a greenhouse in the Plant Sciences Department, University of California, Davis, Davis, CA, United States, under natural daylight (14–15 h light/9–10 h dark), and irrigated daily with deionized water. Shortly after arrival, original media was removed and the plants were transplanted into Stuewe D40H pots (6.4 cm × 25.4 cm) filled with media consisting of perlite:sand:fir bark:peat moss, 3:1:1:1. The roots were inoculated at the time of transplant. No preexisting root nodules were observed in any plants. Soil collected from the rhizosphere of *Ceanothus velutinus* shrubs growing in Sagehen Experimental Forest (Truckee, CA, United States) was used as inoculum. The soil inoculum was directly applied to the exposed root ball.

*Datisca* *glomerata* seeds were collected from wild *D.* *glomerata* plants growing in Gates Canyon, Vacaville, CA, United States. Surface-sterilized seeds were germinated on moistened autoclaved vermiculite in a Magenta^TM^ GA-7 Plant Culture Box, in a controlled environment (25°C, 16 h light/8 h dark). Once seedlings reached approximately 2 cm, they were transplanted into 5 cm × 5 cm × 7 cm pots filled with the same media used for the *C*. *thyrsiflorus* cuttings, and moved to the same greenhouse as the *C.* *thyrsiflorus* cutting. Seedlings were irrigated daily with deionized water.

One-half-strength Hoagland’s solution without nitrogen (Hoagland and Arnon, 1938) was applied twice a week for 9 weeks. The roots were then cut back and repotted with fresh media. The re-grown roots were collected 6 days later, and frozen immediately in liquid nitrogen (see section “*Tissue Sampling*”).

Three days after the roots had been collected, seedlings were inoculated. Crushed nodules were collected from a different set of *D.* *glomerata* seedlings and used as inoculum. These previous seedlings had been inoculated with crushed nodules that originated from the same inoculum source as the *C.* *thyrsiflorus* cuttings described above.

### Tissue Sampling

Roots tips and nodules of both *C. thyrsiflorus* and *D. glomerata* were collected as pairs from the same individual plants: for *C.* *thyrsiflorus*, pairs of root and nodule were collected from three individual plants; for *D.* *glomerata* pairs of root and nodule were collected from 6 individual plants. The *C.* *thyrsiflorus* nodules were collected 75 days or 96 days (11 or 14 weeks) after inoculation, and *D.* *glomerata* nodules were collected 24 days after inoculation. All plants were first placed under a halogen lamp at 250 μEm^-2^s^-1^ for over 1 h to stabilize photosynthesis. For roots, root tips (approximately 2.5 cm) were collected, and for nodules, whole nodules (ranging from single- to multi-lobed) were collected (**Supplementary Image S4**). To remove media particles, roots or nodules were rinsed in deionized water immediately before flash freezing in liquid nitrogen. The sampling process was kept under 5 min per plant. Frozen tissues were stored at -80°C until RNA extraction.

### RNA Extraction, Sequencing, and Transcriptome Assembly

Total RNA was extracted from each sample (see **Supplementary Table S9** for the concentrations and integrity scores for each RNA extracts). Barcode-indexed RNA-seq libraries were prepared in the DNA Technologies and Expression Analysis Cores at the University of California, Davis Genome Center (see **Supplementary Table S10** for the adapter and barcode sequences, see **Supplementary Image S5** for fragment sizes). The libraries were pooled in equimolar ratios for sequencing.

High-throughput sequencing was carried out in the DNA Technologies and Expression Analysis Cores at the UC Davis Genome Center. For *C.* *thyrsiflorus*, the pooled libraries were sequenced on a single lane of Illumina HiSeq 4000 (Illumina, San Diego, CA, United States) platform with paired-end 150 bp reads (PE150). For *D.* *glomerata* RNA-seq, the pooled libraries were sequenced on two lanes of Illumina HiSeq 2500 (Illumina) platform (PE150).

Raw sequence reads were trimmed based on read quality and adapter contamination using Scythe v0.991 (Buffalo, 2014) and Sickle v1.22 (Joshi and Fass, 2011) and the quality of cleaned reads were assessed using FastQC v0.11.2 (Andrews, 2010). Insert sizes were verified using Bowtie2 v2.2.6 (Langmead and Salzberg, 2012), Samtools v1.2 (Li, 2011), and Picard (Nazaire, 2017) (**Supplementary Images S5**, **S6**).

Cleaned high-quality paired-end reads were used to assemble a single root + nodule transcriptome using Trinity v2.20 (Grabherr et al., 2011) for *C.* *thyrsiflorus* and v2.06 for *D.* *glomerata*. The *C.* *thyrsiflorus* transcriptome was assembled on DIAG (Data Intensive Academic Grid) (White et al., 2010), and the *D.* *glomerata* transcriptome was assembled on the UC Davis Bioinformatics Core high-performance computing cluster. The newly assembled raw root + nodule transcriptomes were curated by passing through three independent screenings. In short, transcripts with low (<5) transcripts per million (TPM) in both tissues were removed in Screen-1 using RSEM v1.2.31 (Li and Dewey, 2011), transcripts with none or only short (<298 bp) CDSs were removed in Screen-2 using TransDecoder v3.0.1 (Haas et al., 2013) and ORFfinder (Wheeler et al., 2013), and transcripts with no similar sequence found in GenBank (Benson et al., 2005) non-redundant (nr) database were removed in Screen-3 using BLASTP v2.5.0+ (Altschul et al., 1990) (i.e., sequences with no hits better than 1e-20 were removed). Because neither the *C.* *thyrsiflorus* cuttings nor *D.* *glomerata* seedlings were clonal, alleles were collapsed using Allelepipe v1.0.28 (Dlugosch et al., 2013) (see **Supplementary Data Sheet S1** for full description).

### Validation of RNA-seq With Real-Time Quantitative Reverse Transcription PCR

The assembled sequences and their respective fold changes determined via RNA-seq were validated using real-time quantitative reverse transcription PCR (RT-qRT-PCR). A total of nine genes were selected for RT-qRT-PCR validation in each plant (**Supplementary Table S7**) using orthologs in *D.* *glomerata* and *C.* *thyrsiflorus* determined by OrthoReD (**Supplementary Table S5**): five genes that are universally nodule-enhanced (*NIN, SYMREM, RPG, NRT1.8, CYP735A*), two genes that are universally root-enhanced (*MAX4, AHK5*) according to the RNA-seq results, and two additional reference genes (Ubiquitin ligase, Glyceraldehyde 3-P Dehydrogenase) used as controls. The controls were selected based on the reference genes tested in *Medicago* *sativa* (Castonguay et al., 2015).

Primer-BLAST (Ye et al., 2012) was used to design all PCR primers (**Supplementary Table S7**). Default settings were used except the size of the product was limited to 90–160 bps. PCR primers were chosen to amplify a segment near the middle of the transcript. All PCR primer pairs had a self-complementarity score and self-3′-complementarity score below 8.

Roots and root nodules were sampled from three different individuals of *C.* *thyrsiflorus* and three different individuals of *D.* *glomerata* with the same methods used to sample material for RNA-seq. Here, the nodules of *C.* *thyrsiflorus* and *D.* *glomerata* nodules were 84 days (12 weeks) and 48 days after inoculation, respectively, and the inoculation was about a month earlier in the season compared to the samples used for RNA-seq.

RNA extraction was performed as described in RNA-seq experiments. This was followed by DNAse treatment using Turbo DNA-free kit (Thermo Fisher, CA, San Diego, United States). Efficacy of DNAse treatment was confirmed by negative PCR amplification targeting plastidic *trnL* gene (data not shown). cDNA libraries were constructed using SuperScript^TM^ III First-Strand Synthesis System Kit (Thermo Fisher, CA, United States) following manufacturer’s protocol. RT qRT-PCR was performed using VeriQuest Fast SYBR Green qPCR Master Mix (Thermo Fisher, CA, United States) following manufacturer’s protocol on a 7500 Fast Real-Time PCR system (Thermo Fisher, CA, United States).

Once the threshold cycle (*C*_T_) was determined for each reaction, the average *C*_T_ for each gene was calculated for each tissue for each species. Then the *C*_T_ values for the seven target genes (*NIN, SYMREM, RPG, NRT1.8, CYP735A, MAX4, AHK5*) were normalized using the *C*_T_ of the reference. The nodule/root log2-fold change was calculated as the difference between the normalized *C*_T_ values of nodules and roots. This was compared to the nodule/root log2-fold fold change estimated based on RNA-seq.

### *M. truncatula* Transcriptome and Genome

Published root and nodule (15 days after inoculation) transcriptome data were obtained from a previous study (Roux et al., 2014). The genome sequence of *M.* *truncatula*, i.e., the full length CDSs of 50,894 genes, was obtained from Phytozome v11.0 (Goodstein et al., 2012) (**Supplementary Table S3**). For genes that were not expressed in either of the tissues, the expression levels, the log2-fold changes, and the associated *p*-values were considered 0, 0, and 1, respectively. For genes that were tissue specific, the log2-fold changes were set as 15 (+15 for nodule-specific, -15 for root-specific genes), and the associated *p*-values were set as 1 whenever the values were not already provided. Henceforth, *M.* *truncatula* genome will refer to all the genes within the genome while *M.* *truncatula* transcriptome will refer to the subset (28,260 genes) that is expressed in the roots and/or in the nodules.

### Transcriptome and Genome Annotation

The *C.* *thyrsiflorus* and *D.* *glomerata* transcriptomes and the *M.* *truncatula* genome were annotated using InterProScan v5.21 (Jones et al., 2014) (options: -goterms -pa) and Trinotate v3.0.1 (Haas et al., 2013) (options: -E 1e-5 –pfam_cutoff DNC) in parallel, to provide transcript annotations according to KEGG orthology (KO), Enzyme commission (EC) number, InterPro ID, Pfam ID, EggNOG ID, GO ID. The *M. truncatula* genome was previously annotated (Roux et al., 2014), but was reannotated to have consistency across all three species.

### Transcriptome and Genome Completeness

Two analyses were conducted in parallel to assess and compare the completeness of the three root + nodule transcriptomes and *M.* *truncatula* genome. BUSCO v2.0 with Embryophyte reference (Simao et al., 2015) (options: -m proteins -sp arabidopsis) was used to estimate the completeness based on the fraction of plant-universal single-copy orthologs found in each transcriptome or genome. Each transcriptome or genome was also passed through the KEGG-KAAS server (Moriya et al., 2007) as amino acids and used as a query in searches against all plant genomes via BLAST and annotated based on single-directional best hits (SBHs), to determine how many enzymes in terms of EC number are present within each biosynthetic pathway listed in KEGG.

### Differential Gene Expression Analysis

Analyses of differential gene expression between the two tissues (nodule and root) for *C.* *thyrsiflorus* (*n* = 3) and *D.* *glomerata* (*n* = 6) were conducted using R v3.3.1 (R Core Team, 2013) with limma v3.3.3 Bioconductor package (Ritchie et al., 2015). Expected reads for each gene were estimated by using RSEM v1.2.31 (Li and Dewey, 2011) based on the mapping results of cleaned reads on to the root and the nodule transcriptomes and were used as the basis for the differential gene expression analysis. For *M.* *truncatula* (*n* = 3), the gene expression levels in the root and in the nodule, nodule/root log2-fold change, and associated adjusted *p*-values were obtained from a previous study (Roux et al., 2014). Genes with the log2-fold change over ± 1 and *p*-values (adjusted for repeated measures) of <0.005 for *C.* *thyrsiflorus* or <0.001 for *D.* *glomerata* and *M.* *truncatula* were considered to represent significantly differential expression. The *p*-value threshold for *C.* *thyrsiflorus* was set higher than the other two plants because limma did not generate any *p*-values < 0.001 due to less statistical power as a result of the fewer biological replicates for each tissue compared to *D.* *glomerata*.

Gene Ontology enrichment analysis (Gene Ontology Consortium, 2017) was conducted on both the root- and nodule-enhanced genes against the root + nodule transcriptome to detect GO terms and corresponding functions enriched in the root and the nodule. GO enrichment analysis was conducted using R v3.3.1 with topGO v2.28.0 Bioconductor package (Alexa and Rahnenfuhrer, 2016) based on gene counts of each GO term using runTest() (options: algorithm = ”classic”, statistic = ”fisher”). After calculating the raw *p*-values, to account for multiple testing, the significance of each GO term was tested through Benjamini–Hochberg procedure. The *p*-value thresholds was set at α = 0.01, and the false discovery rate was set at *Q* = 0.20.

### Database for Orthology Comparison

To enable the comparison of orthologous sets of genes across and beyond the NFC, a nucleotide sequence database was constructed with 12 genomes (total CDSs) from species within the NFC and close outgroups in combination with the *C.* *thyrsiflorus* and *D.* *glomerata* transcriptomes and the *M.* *truncatula* genome (see **Supplementary Table S11** for the complete list). These sequences were collected from three different databases: Phytozome v11 (Goodstein et al., 2012), Genome Explorer (Carleton College, 2010), and miyakogusa.jp 3.0 (Sato et al., 2008). A small fraction (<0.1%) of the CDSs originally collected could not be translated reliably due to ambiguous reading frames or premature stop codons. These sequences were removed from the analyses.

### Orthology Predictions

Orthologs for each gene in the *C.* *thyrsiflorus* and *D.* *glomerata* transcriptomes and the *M.* *truncatula* genome were predicted independently using OrthoReD (Battenberg et al., 2017) (options: –blast_type NCBI –loci_threshold 10 –sander_schneider YES –overlap_threshold 0) with *Vitis* *vinifera* (Vitaceae, Vitales) set as the outgroup. In order to capture orthologs beyond the in-paralogs (Sonnhammer and Koonin, 2002) expected from the genome duplications in Fabaceae (Cannon et al., 2010) and Rosaceae (Potter et al., 2007), OrthoReD was modified to ignore gene duplication(s) within a single order (Customized version of OrthoReD^1^). For genes with multiple isoforms or alleles, orthologs were predicted independently for each member, and the union of the predictions of all isoforms was considered the set of predicted orthologs for the gene. The predicted orthologs were then merged into 27,367 non-overlapping MergedOrthoGroups using a custom Perl script (**Supplementary Table S5**).

In addition, orthologs of 19 genes from *M.* *truncatula* or *L.* *japonicus* whose functions in the establishment of RNS have been well characterized (Kouchi et al., 2010; Oldroyd, 2013) were also predicted (see **Table 1** for the gene list). Reference sequences for these 19 RNS pathway genes were collected from GenBank, and verified in the database assembled in this study. OrthoReD with the same parameter settings as other sequences were used to predict orthology for *L.* *japonicus* genes.

### Presence of RNS Pathway Genes

For each of the 19 RNS pathway genes, a set of genes was collected comprising every *C. thyrsiflorus* or *D.* *glomerata* gene that was (1) predicted as a putative ortholog starting with the reference gene as a query, and/or (2) predicted the reference gene as its putative ortholog. The presence/absence of orthologs for each gene identified as a part of the RNS pathway established in *M.* *truncatula* and/or *L.* *japonicus* was scored for each newly assembled transcriptome and compared to previous studies published on *Alnus* *glutinosa* and *Casuarina* *glauca* (Hocher et al., 2011), and *D.* *glomerata* (Demina et al., 2013).

### Expression Similarity Analysis

From the 27,367 MergedOrthoGroups, we first parsed out whole MergedOrthoGroups, each of which: (1) contained at least one member from each of *C.* *thyrsiflorus, D.* *glomerata*, and *M.* *truncatula*; and (2) was identical to a set of orthologs predicted from a single gene. We also parsed subsets within the remaining MergedOrthoGroups that met the aforementioned two criteria only with respect to the three species *C.* *thyrsiflorus, D.* *glomerata*, and *M.* *truncatula*.

Then the gene expression pattern was scored as root-enhanced, nodule-enhanced, or not significantly different between the two tissues based on the differential gene expression analysis for each species for each MergedOrthoGroup. For MergedOrthoGroups where a single species was represented by more than one paralog, the gene expression pattern for this species was considered root- or nodule-enhanced if there was at least one significantly differentially expressed paralog, unless one or more of them were enhanced in one tissue and other paralog(s) were enhanced in the other tissue, in which case the MergedOrthoGroup was considered to be both root- and nodule-enhanced for that species. Among the representative MergedOrthoGroups, those that were either nodule-enhanced or root-enhanced in all three species were collected and designated as the core MergedOrthoGroups.

In order to determine the functions of RNS establishment that are conserved across the three species, root- and nodule-enhanced genes within the core MergedOrthoGroup were collected separately for each species, and GO enrichment analysis was conducted on these genes against their respective root + nodule transcriptome using the same conditions as described above.

Next, we tested how strongly the differences in gene expression between roots and nodules in each species are correlated with those of other species using the approach previously applied for measuring the similarities between biological replicates (Kempema et al., 2007). For each pair of species, the gene expression level fold changes between the two tissues were plotted for all MergedOrthoGroups. The Pearson correlation coefficient was then calculated separately for all MergedOrthoGroups, for MergedOrthoGroups that were nodule-enhanced in both species, and for those that were root-enhanced in both species using cor.test() available in R v3.3.1, stats package v3.3.1. For MergedOrthoGroups where at least one of the species was represented by more than one paralog, the most similar pair of fold changes was used as the representative.

Finally, the degree and significance of overall similarity in gene expression between each pair of species was assessed using dissonance scores (summarized in **Supplementary Image S2**). For each pair of species for each MergedOrthoGroup, the degree of dissonance between the species was calculated as the average pairwise difference in fold change between all transcripts for the two species belonging to that group (for some groups, one or more species was represented by more than one transcript). For example, the dissonance score (*D*) between two species A and B for a given MergedOrthoGroup each with *m* and *n* representative transcripts, was calculated as $D=\left[\sum_{i=1}^{m}\left(\sum_{j=1}^{n}\left|A_{i}-B_{j}\right|\right)\right]/(m*n)$. Next, the overall dissonance score between species A and B was calculated as the total sum of the dissonance scores of all representative MergedOrthoGroups. Finally, in order to determine the statistical significance of this measure, a permutation test (random resampling of fold change values with no replacement) was conducted to calculate the overall dissonance score for 10,000 replicates, and the *p*-value was calculated as the fraction of permutations that yielded an overall dissonance score equal to or smaller than the observed data.

The normal distributions of the total dissonance scores among the permutations were verified using R v3.3.1 with stats package v3.3.1 based on quantile-quantile plots and histograms generated using qqnorm() and hist() (**Supplementary Image S7**).

### *dN/dS* Analysis

The aforementioned 3,894 MergedOrthoGroups, i.e., sets of orthologs that (1) contained at least one member from each of *C.* *thyrsiflorus, D.* *glomerata*, and *M.* *truncatula*, and (2) were identical to a set of orthologs predicted from a single gene, were used in this analysis.

The three competing hypotheses (single-origin, multiple-origin, and two-step hypothesis) differ in the expected timing(s) of gaining predisposition or function of RNS, the events that would have caused changes in selection pressures on the genes involved. A change in selection pressure can be detected as a change in the average ratio of non-synonymous to synonymous mutations (*dN*/*dS*) (Hurst, 2002).

For each MergedOrthoGroup, the likelihoods of up to three different hypothesis-based scenarios (SINGLE, MULTI, and TWOSTEP), each of which predicts a different timing for a change in *dN*/*dS*, were compared to a NULL scenario, which assumes a constant *dN*/*dS* throughout the gene phylogeny (**Figure 4**). The SINGLE scenario, based on the hypothesis of a single origin of RNS, assumes a different *dN*/*dS* along the branch leading to the base of the NFC (branch *a* in **Figure 4**) compared to the rest of the tree. The MULTI scenario, based on the hypothesis of multiple independent origins of RNS, assumes different values of *dN*/*dS* along the branches leading to *C*. *thyrsiflorus, D.* *glomerata*, and the legumes, (branches *b, c*, and *d*, respectively, in **Figure 4**) compared to the rest of the tree. The TWOSTEP scenario, based on the hypothesis of a gain of predisposition to RNS at the base of the NFC followed by independent gains of function in each lineage exhibiting RNS, assumes different values of *dN*/*dS* for all of the aforementioned branches (*a* through *d* in **Figure 4**) compared to the rest of the tree.

To serve as the basis for comparisons between these scenarios, a multiple sequence alignment (MSA) and a most-likely gene phylogeny were generated for each MergedOrthoGroup. Each MSA was generated using MAFFT v7.272 (Katoh and Standley, 2013) (options: –amino –localpair –retree 2 –maxiterate 1000). Two phylogenetic trees were constructed using RAxML v8.2.8 (Stamatakis, 2006) (options: -f d -m GTRGAMMAIX). The topology of one tree was constrained to the family level according to the established phylogeny (APG, 2009) (see **Figure 4** as a reference), while the topology of the second tree was not constrained. For each tree, four parallel searches were carried out to prevent lodging on to local optima. To select one of the two trees for further analysis, the two trees were compared using a K-H test (Kishino and Hasegawa, 1989) through PAML v4.8 (Yang, 2007) (options: codeml, clock = 0, aaDist = 0, model = 0, NSsites = 0, fix_blength = -1). Unless the unconstrained tree was significantly better than the constrained tree (*p* < 0.05) the constrained tree was used for further analyses.

For each MergedOrthoGroup, the MSA was fitted onto the most-likely tree under the four scenarios to calculate the likelihood scores for each using PAML v4.8 (options: codeml, clock = 0, aaDist = 0, model = 2, NSsites = 0, fix_blength = -1). An S-H test (Shimodaira and Hasegawa, 1999) was carried out simultaneously in PAML. Scenarios significantly worse than the best (*p* < 0.05) were rejected, and MergedOrthoGroups that did not reject the NULL scenario were assumed to conform to the NULL scenario. In some MergedOrthoGroups for which the unconstrained topology was used, some scenarios were not tested because the clade required for the scenario was missing from the tree.

### Hypothesis Testing

Based on the results generated by the methods described above, the three competing hypotheses (single-origin, multiple-origin, and two-step) were tested against each other. The multiple-origin hypothesis differs from the other two hypotheses in that it assumes completely independent gains of RNS in different lineages. To test this hypothesis, we used the presence/absence of RNS pathway genes in the two actinorhizal plants, and the gene expression patterns from the three different lineages for each orthologous set of genes, to see if the three lineages were significantly more similar to one another than expected by random chance (see section “*Expression Similarity Analysis”* under Material and Methods). A significant result would indicate the presence of a common evolutionary background among the three species compared (which are phylogenetically distantly related species distributed throughout the NFC), supporting single-origin or two-step hypothesis, and rejecting the multiple-origin hypothesis.

The *dN*/*dS* analysis directly compares the three hypotheses. Among MergedOrthoGroups that rejected the NULL scenario, MergedOrthoGroups that fail to reject only the MULTI scenario would be consistent with both the multiple-origin hypothesis and the two-step hypothesis, because the two-step hypothesis does not require the same gene to be the cause of both the gain-of-predisposition and the gain-of-function. However, such genes would be in conflict with the single-origin hypothesis because additional changes for each RNS host lineage are not assumed in this hypothesis. Likewise, genes that fail to reject only the SINGLE scenario would be consistent with either the single-origin hypothesis or the two-step hypothesis, but would be in conflict with the multiple-origin hypothesis. Finally, genes that fail to reject only the TWOSTEP scenario would be in conflict with both the multiple-origin and single-origin hypotheses, and would only be consistent with the two-step hypothesis.

## Author Contributions

KB conducted the experiments, analyzed the data, and substantially wrote the manuscript. CT conducted the RT-qPCR reactions together with KB. JC, DP, and AB provided suggestions on the experimental design, interpretations for comparative transcriptomics, phylogenetics, and the biology of RNS, and contributed to editing the manuscript. AB provided an initial project framework.

## Conflict of Interest Statement

The authors declare that the research was conducted in the absence of any commercial or financial relationships that could be construed as a potential conflict of interest.

## Footnotes

1 https://github.com/kbattenb/OrthoReD_vNFC

## Abbreviations

ABA: abscisic acid
AM: arbuscular mycorrhizal
CDS: coding sequence
*dN*/*dS*: ratio of non-synonymous to synonymous mutations
IAA: indole-3-acetic acid
MRCA: most recent common ancestor
NFC: nitrogen-fixing clade
NO: nitric oxide
RNS: root nodule symbiosis
