# Supplementary figures and images for: Comparative Transcriptomic Analysis of Two Actinorhizal Plants and the Legume Medicago truncatula Supports the Homology of Root Nodule Symbioses and Is Congruent With a Two-Step Process of Evolution in the Nitrogen-Fixing Clade of Angiosperms
Source: Front Plant Sci. 2018 Oct 8;9:1256. doi: 10.3389/fpls.2018.01256 (PMC6187967; doi:10.3389/fpls.2018.01256)

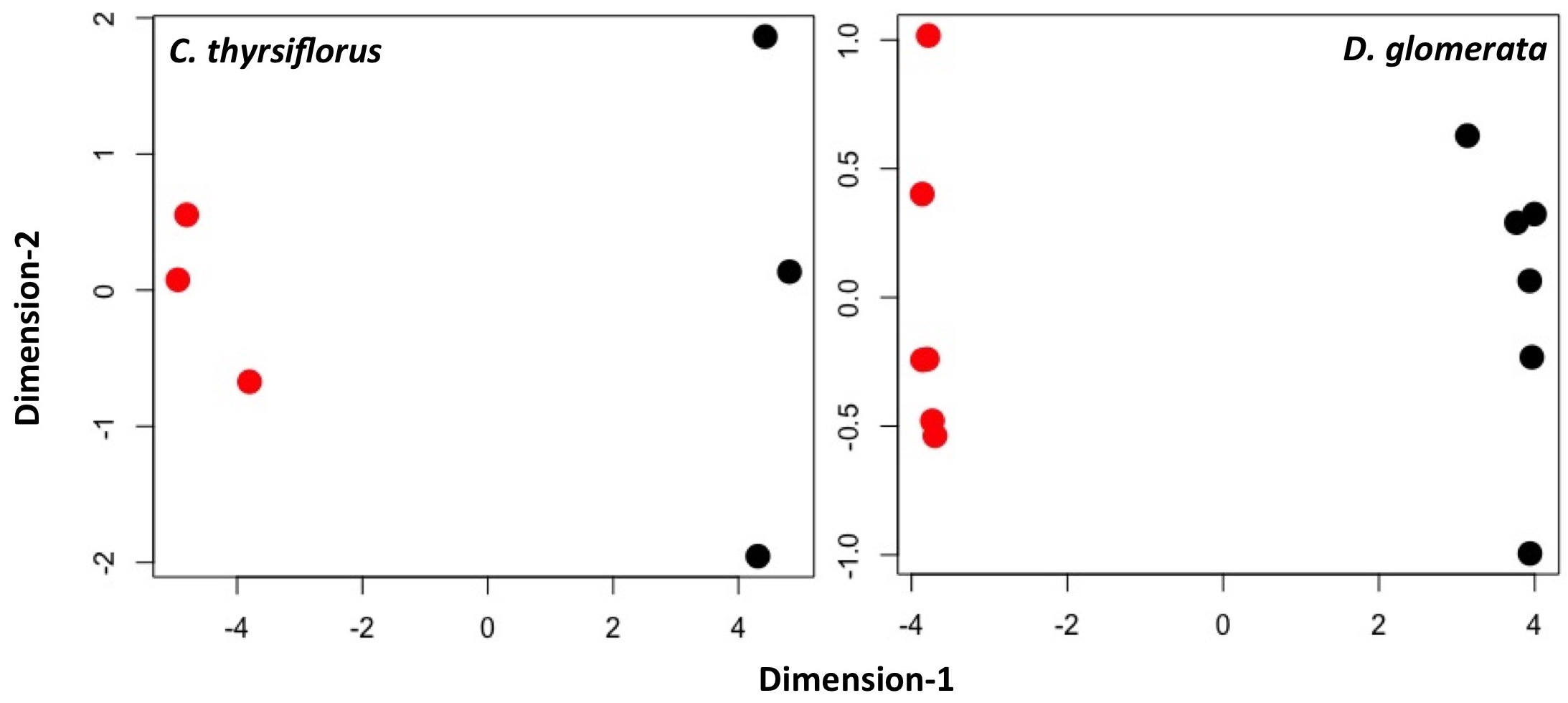

Supplement: Supplementary file 12 [file Image_1.TIF]

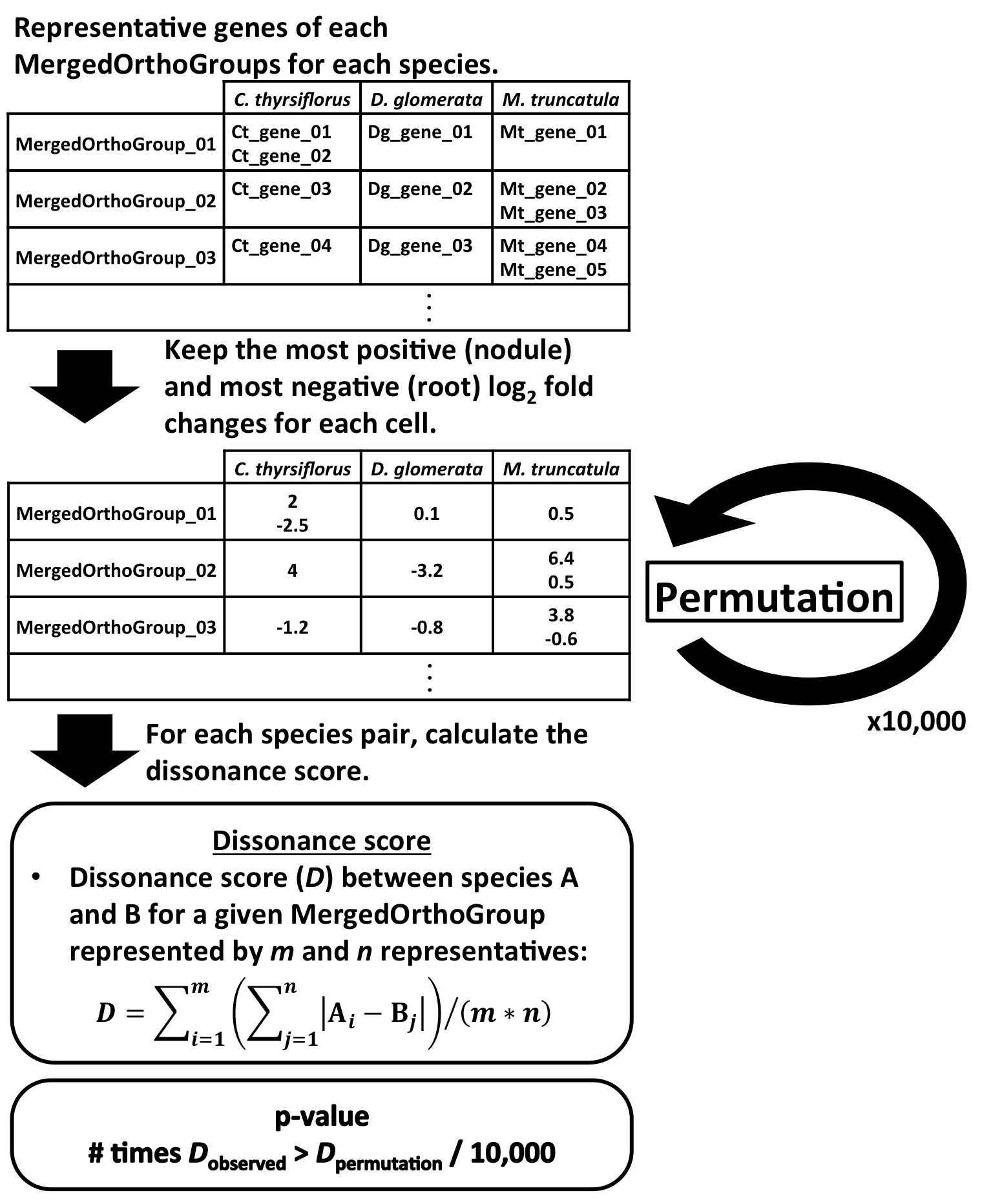

Supplement: Supplementary file 13 [file Image_2.TIF]

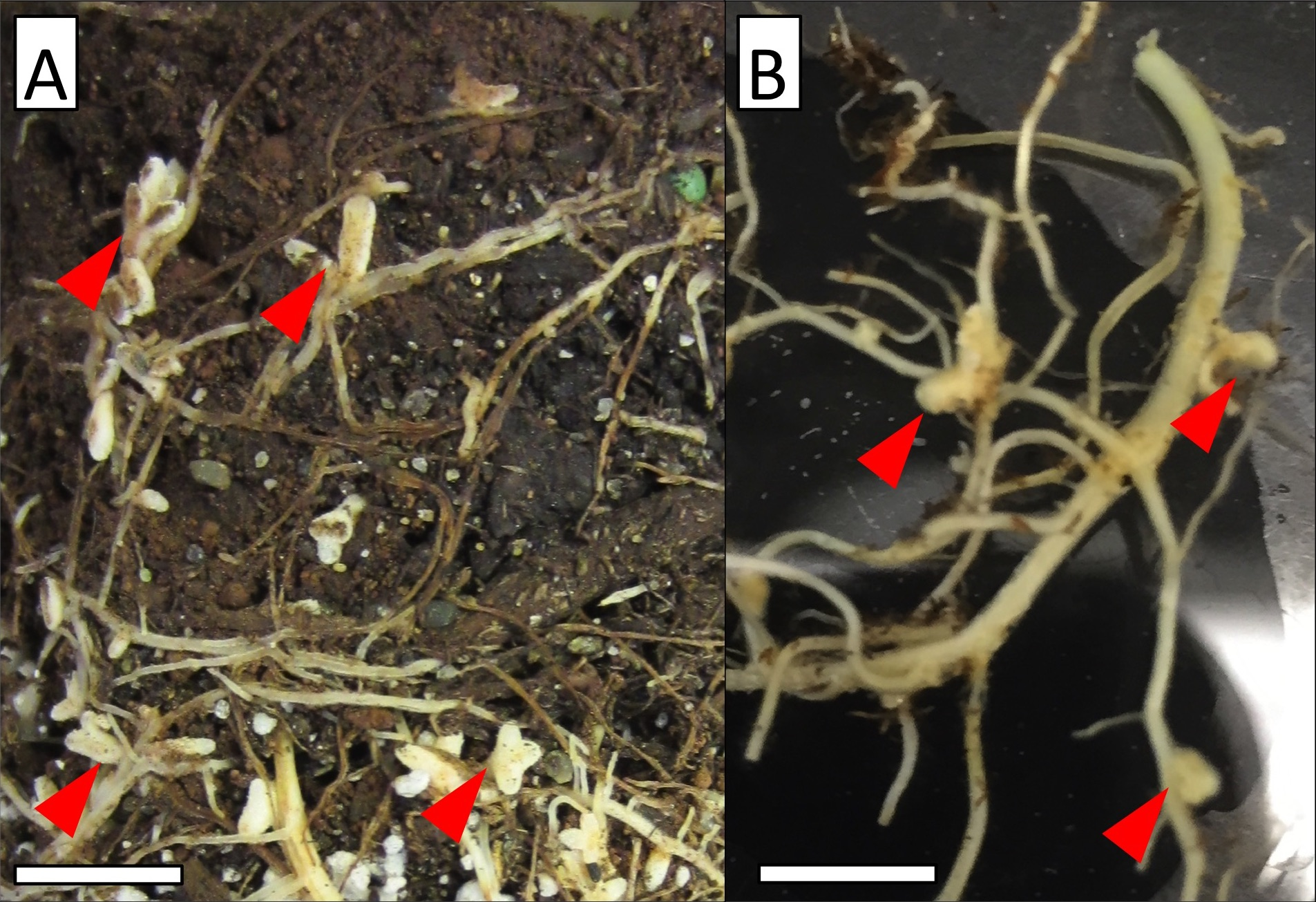

Supplement: Supplementary file 15 [file Image_4.TIF]

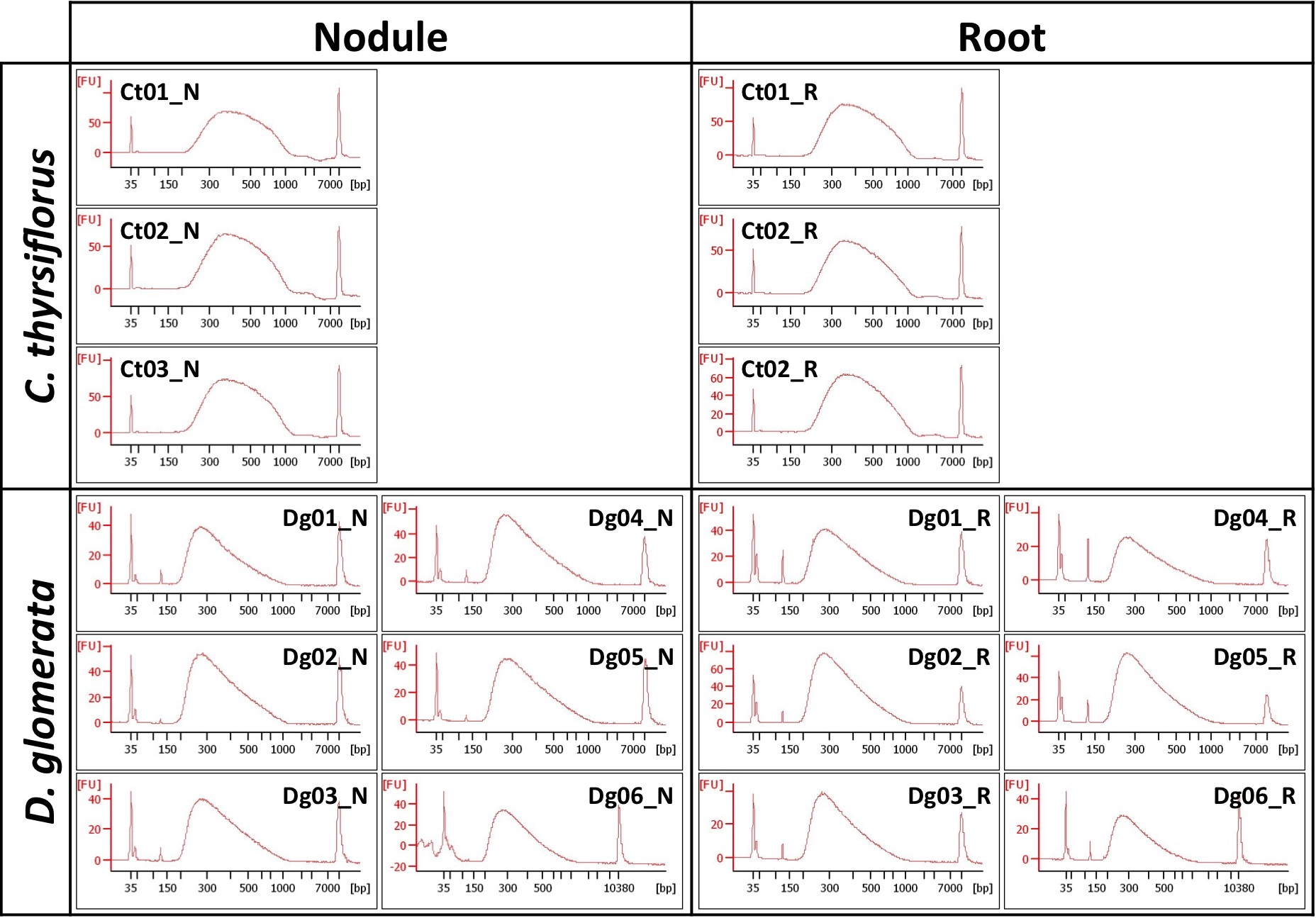

Supplement: Supplementary file 16 [file Image_5.TIF]

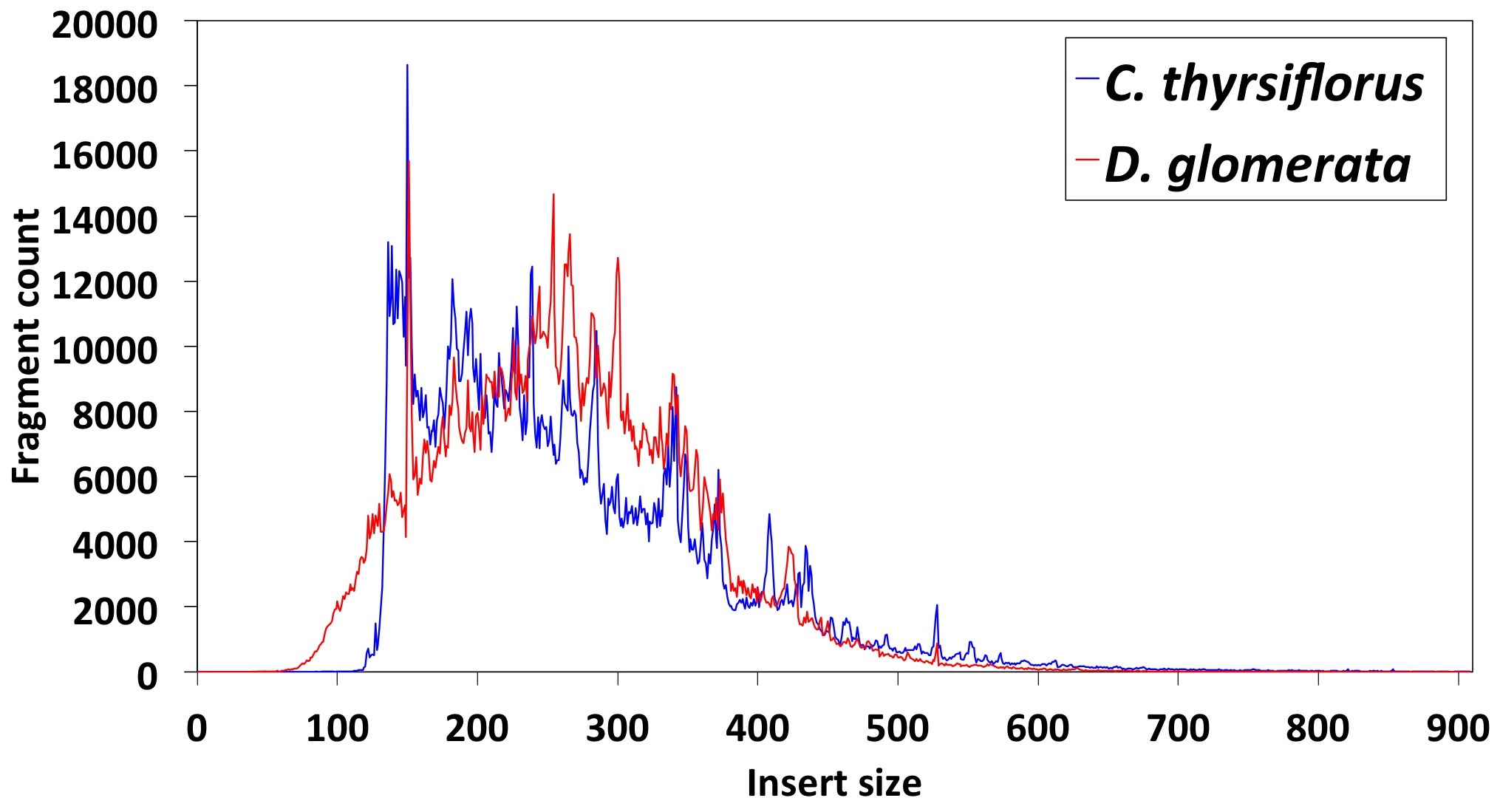

Supplement: Supplementary file 17 [file Image_6.TIF]

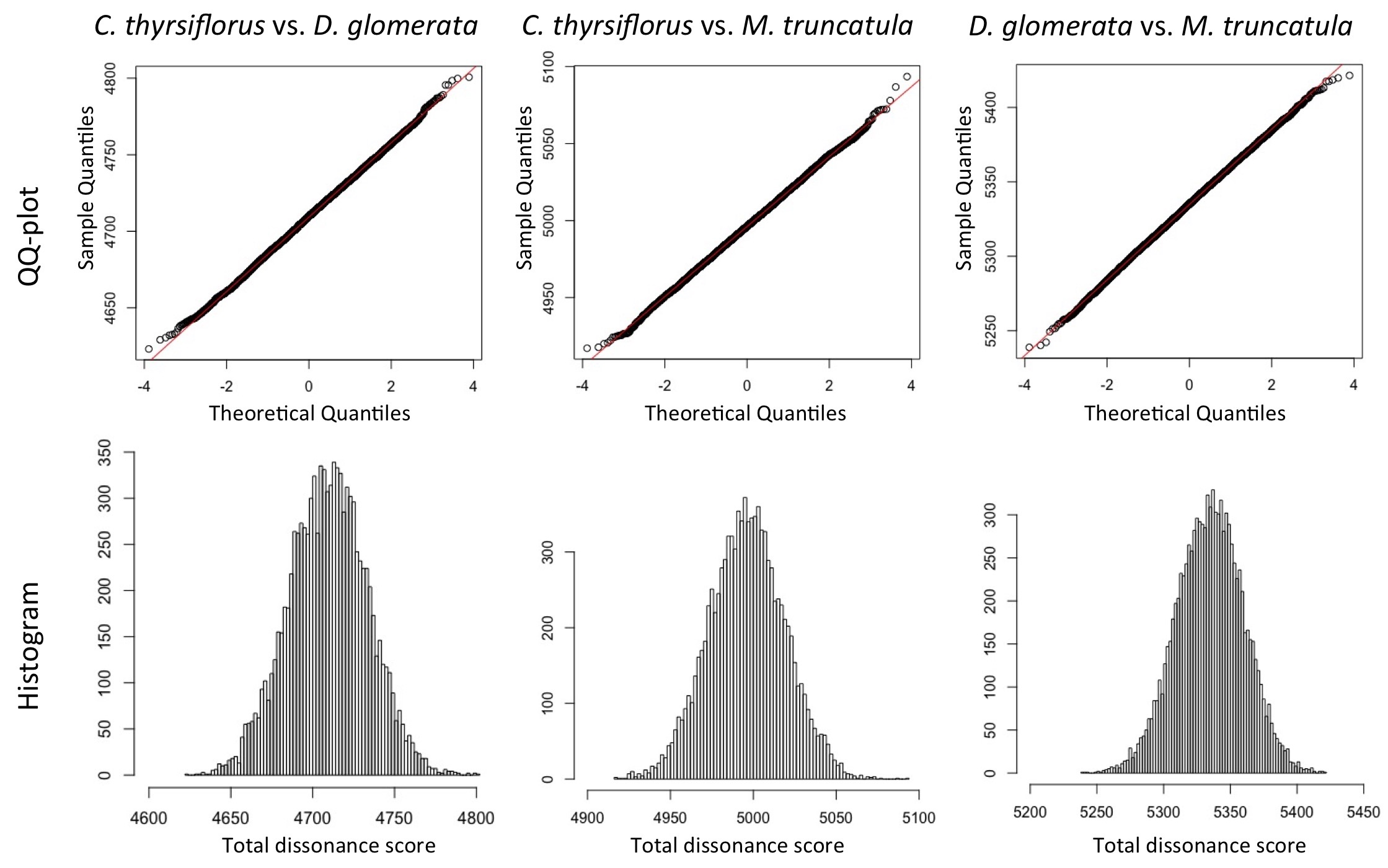

Supplement: Supplementary file 18 [file Image_7.TIF]
