# Supplementary material for: Comparative Transcriptomic Analysis of Two Actinorhizal Plants and the Legume Medicago truncatula Supports the Homology of Root Nodule Symbioses and Is Congruent With a Two-Step Process of Evolution in the Nitrogen-Fixing Clade of Angiosperms
Source: Front Plant Sci. 2018 Oct 8;9:1256. doi: 10.3389/fpls.2018.01256 (PMC6187967; doi:10.3389/fpls.2018.01256)
Supplement: Supplementary file 19 [file Data_Sheet_1.docx]

***RNA extraction and library preparation***

Collected tissues were ground into a fine powder in liquid nitrogen, using mortar and pestle, and prepared for RNA-seq. For *C*. *thyrsiflorus*, total RNA was extracted using the Spectrum™ Plant Total RNA Kit (Sigma-Aldrich, CA, USA) according to the manufacturer’s protocol, and for *D.* *glomerata*, total RNA was extracted using RNeasy Plant Mini Kit (Qiagen, CA, USA) according to the manufacturer’s protocol. Additionally, off-column DNA digestion was performed on all RNA extracts, using the TURBO DNA-free™ Kit (ThermoFisher, CA, USA) according to the manufacturer’s protocol. Prior to library preparation, the concentration and the integrity of the extracted RNA were measured using Experion™ Automated Electrophoresis System to ensure that high-quality RNA extracts were used for library preparation. For *C.* *thyrsiflorus*, pairs of root and nodule RNA extracts from 3 individual plants were selected for library construction; for *D.* *glomerata* pairs of root and nodule extracts from 6 individual plants were selected for library construction (See Supplemental Table S1 for the concentrations and integrity scores for each RNA extracts).

Library preparations were carried out in the DNA Technologies and Expression Analysis Cores at the University of California, Davis Genome Center. Barcode-indexed RNA-seq libraries were generated from 500ng of total RNA each after poly-A enrichment using the Kapa Stranded RNA-seq kit (KapaBiosystems, Cape Town, South Africa) according to the manufacturer’s protocol. For *D.* *glomerata* samples, the libraries were generated on a Sciclone NGS G3 liquid handler (Caliper Life Sciences, Alameda, CA). Libraries for *C.* *thyrsiflorus* were generated manually. (See Supplemental Table S4 for the adapter and barcode sequences.) Libraries were analyzed with a Bioanalyzer 2100 instrument (Agilent, Santa Clara, CA), and quantified by fluorometry on a Qubit instrument (LifeTechnologies, Carlsbad, CA) to confirm that fragment sizes were suitable (ca. 300bp) for subsequent sequencing (Figure S4). The barcode-indexed libraries were then pooled in equimolar ratios and quantified by qPCR with a Kapa Library Quant kit (KapaBiosystems).

***Sequencing***

High throughput sequencing was carried out in the DNA Technologies and Expression Analysis Cores at the UC Davis Genome Center. For *C.* *thyrsiflorus*, the pooled libraries were sequenced on a single lane of Illumina HiSeq 4000 (Illumina, San Diego, CA) platform with paired-end 150bp reads (PE150). For *D.* *glomerata* RNA-seq, the pooled libraries were sequenced on two lanes of Illumina HiSeq 2500 (Illumina) platform (PE150).

***Transcriptome assembly and cleaning***

Sequenced raw reads were screened and only high-quality sequences were kept for transcriptome assembly. The raw reads were first screened for contamination: Poly A, poly T, or adapter sequences (and the 3’ downstream portion of these contaminations) were trimmed first using Scythe v0.991 (Buffalo, 2014) (options: --min-match 0 --min-keep 35). Reads less than 35bp after trimming were removed. The adapter-trimmed reads were screened for sequencing quality: The 3’ downstream portion of the read with a Phred quality score <20 was trimmed using Sickle v1.22 (Joshi & Fass, 2011) (options: sickle pe -q 20 -l 35). Reads less than 35bp after trimming were removed. At this point, any paired-end reads with their counterpart removed were removed entirely from the following process. In addition, due to low Phred quality score, the first base (5’ end) of all reads of *C.* *thyrsiflorus* was trimmed. For the raw reads and after each screening step, the overall quality of the reads was assessed using FastQC v0.11.2 (Andrews, 2010). The consistency of the insert sizes and the fragment sizes was confirmed by measuring the insert sizes of the cleaned reads that mapped onto the 18S rRNA sequences of *Ceanothus* *sanguineus* (*C.* *thyrsiflorus* was not available) or *D.* *glomerata* (GenBank reference IDs are U42799.1 and U42426.1 respectively). Reads were mapped using Bowtie2 v2.2.6 (Langmead & Salzberg, 2012) and Samtools v1.2 (Li, 2011), and insert sizes were measured using Picard (Nazaire, 2017) (option: picard.analysis.CollectInsertSizeMetrics) (Supplemental Figures S4, S5).

For each species, all the resulting high-quality paired-end reads were used to assemble a single root+nodule transcriptome using Trinity v2.20 (Grabherr et al., 2011) for *C.* *thyrsiflorus* and v2.06 for *D.* *glomerata* (options: --normalize_reads --seqType fq --min_contig_length 200). The *C.* *thyrsiflorus* transcriptome was assembled on DIAG (Data Intensive Academic Grid) (White et al., 2010), and the *D.* *glomerata* transcriptome was assembled on the UC Davis Bioinformatics Core high-performance computing cluster.

The newly assembled raw root+nodule transcriptomes were curated by passing through three independent screenings. At each stage (including the raw transcriptome), all cleaned reads were mapped onto each transcriptome using Bowtie2 v2.2.6 and Samtools v1.2, and the overall mapping quality was assessed using additional tools included as a part of the Trinity package. Trinity assembles and outputs a list of transcripts that are clustered into genes each with one or more isoforms. Thus, some screening processes were conducted at a gene level, while others were conducted at a transcript level.

Based on the mapping of the cleaned reads onto the raw transcriptome, the expected read counts and transcripts per million (TPM) for each transcript were calculated using RSEM v1.2.31 (Li & Dewey, 2011). TPM of a gene was calculated as the sum of TPMs of all isoforms within each gene. Genes with <5 average TPM or isoforms with <20% average representation within a gene in both tissues (root and nodule) were removed as artifacts of assembly (Screen-1). For each transcript passing Screen-1, the coding sequence (CDS) was predicted using both TransDecoder v3.0.1 (Haas et al., 2013) (options: TransDecoder.LongOrfs -m 100 -G universal -S) and ORFfinder (Wheeler et al., 2013) (options: -c f -g 1 -ml 300 -n t) in parallel. Transcripts that did not contain any putative CDS >=298bp were removed (Screen-2). BLASTP search (Altschul et al., 1990) was conducted using BLAST v2.5.0+ (options: -evalue 1e-20 -word_size 7) on all translated putative CDSs of all transcripts passing Screen-2 against a partial RefSeq non-redundant protein database (nr database) from GenBank (Benson et al., 2005), which only contained sequences from Angiosperms and *Frankia* [Accessed on Oct. 12, 2016]. Only the transcripts with at least one CDS that had a hit to an Angiosperm sequence with an e-value <=1e-20 were kept (Screen-3). When multiple coding sequences (CDSs) were identified for a given transcript, only the one with the best e-value was kept as the CDS of the transcript. For genes with multiple isoforms, the longest CDS among them was set as the representative sequence, which was used for any analysis conducted at a gene level. Additionally, among the removed transcripts, those containing CDSs that had a hit to a *Frankia* sequence with an e-value <=1e-20 were kept as putative *Frankia* transcripts.

Because neither the *C.* *thyrsiflorus* cuttings nor *D.* *glomerata* seedlings were clonal, it was possible for each transcriptome at this point to contain two or more alleles of some genes. To account for this, Allelepipe v1.0.28 (Dlugosch et al., 2013) (options: -p 95 -e 90 -a 300 -m 26 -l M -g 2.0 -c D -x 2) was used to collapse any sequences that shared >=95% identity for a range of >=300bp as different alleles of a single gene. The longest representative sequence of all the alleles was chosen as the representative sequence of the newly collapsed gene, and the expression levels (TPMs) of such genes were calculated as the sum of all alleles. No genes or transcripts were removed in this specific step. Finally the root+nodule transcriptome was separated into root and nodule transcriptomes.
