# Supplementary material for: Comparative Transcriptomic Analysis of Two Actinorhizal Plants and the Legume Medicago truncatula Supports the Homology of Root Nodule Symbioses and Is Congruent With a Two-Step Process of Evolution in the Nitrogen-Fixing Clade of Angiosperms
Source: Front Plant Sci. 2018 Oct 8;9:1256. doi: 10.3389/fpls.2018.01256 (PMC6187967; doi:10.3389/fpls.2018.01256)
Supplement: Supplementary file 20 [file Data_Sheet_2.docx]

***Supplementary Material***

**Comparative Transcriptomic Analysis of Two Actinorhizal Plants and the Legume *Medicago* *truncatula* Supports the Homology of Root Nodule Symbioses and Is Congruent With a Two-step Process of Evolution in the Nitrogen-Fixing Clade of Angiosperms**

**Kai Battenberg^1*^, Daniel Potter^1^, Christine A. Tabuloc^2^, Joanna C. Chiu^2^, Alison M. Berry^1^**

^1^Department of Plant Sciences, University of California, Davis, Davis, CA, United States

^2^Department of Entomology and Nematology, University of California, Davis, Davis, CA, United States

*** Correspondence:**

Kai Battenberg

[kbattenberg@ucdavis.edu](mailto:kbattenberg@ucdavis.edu)

**Figure S1. Multi-dimensional scaling plots of transcriptomes.**

Multi-dimensional scaling (MDS) plots based on the gene expression patterns of the two transcriptomes. Red dots represent nodule samples, and black dots indicate root samples. The nodule and root samples are separated according to Dimension-1 in both cases.

**Figure S2. Scheme of the expression similarity analysis.**

**Figure S3. Enriched GO terms in the core MergedOrthoGroups of *M.* *truncatula*.**

GO terms enriched among the nodule-enhanced (A) and root-enhanced (B) core MergedOrthoGroups, relative to a corresponding root+nodule transcriptome (*M.* *truncatula* depicted as an example). Each circle or box represents a specific GO term with the p-value for significant enrichment indicated below. The lower the p-value for the GO term enrichment, the darker the shade: pale yellow, orange, and red. The top 15 GO terms with the most significant p-values are indicated in a box instead of a circle.

**Figure S4. Examples of roots and root nodules collected for transcriptomes.**

Roots and nodules of *Ceanothus* *thyrsiflorus* (A) and *Datisca* *glomerata* (B) are shown. Root nodules (indicated by the red arrows) of similar age were sampled for the experiment. Scale markers represent 1cm.

**Figure S5. Library fragment sizes of each sample.**

**Figure S6. Insert sizes of transcriptomes.**

Insert size assessment based on the mapping of reads on 18S rRNA sequences. *C.* *thyrsiflorus* and *D.* *glomerata* are indicated in blue and red respectively.

**Figure S7. QQ-plot and histogram of total dissonance score among the permutations for each pairwise comparison.**

**Table S1. Read counts.**

**Table S2. Assembly statistics.**

**Table S3. BUSCO detection.**

T: Transcriptome

G: Genome

**Table S4. Enzymes in all the KEGG biosynthetic pathways represented in transcriptomes and *M.* *truncatula* genome.**

The pathways that differed in their enzyme counts by three or more are highlighted in yellow.

**Table S5. Annotations, differential gene expression, and orthology predictions for *C.* *thyrsiflorus* and *D.* *glomerata* transcriptomes, and *M.* *truncatula* genome (reannotated).**

a: *C. thyrsiflorus* transcriptome.

b: *D.* *glomerata* transcriptome.

c: *M.* *truncatula* genome (reannotated).

**Table S6. Enriched GO terms in roots and root nodules for the transcriptomes of *C.* *thyrsiflorus*, *D.* *glomerata*, and *M.* *truncatula*.**

**Table S7. RT-qRT-PCR results and corresponding primer sequences.**

The yellow cells indicate that the RT-qRT-PCR results were >2 log2-fold change different from the results expected from RNA-seq. The red cell indicates that the RT-qRT-PCR results and the RNA-seq results are in conflict.

**Table S8. MergedOrthoGroups used for expression similarity analysis.**

a: Genes representing each MergedOrthoGroup for each species.

b: Gene expression pattern for each MergedOrthoGroup for each species.

**Table S9. Quality of total RNA extracts.**

Sample ID: Biological replicates used for the construction of transcriptomes.

RQI: RNA Quality Indicator.

**Table S10. Adapter and barcode sequences of each sample.**

The XXXXXX included in the index adapter represents the barcode sequences.

**Table S11. Genomes and transcriptomes used for NFC-wide analyses.**

**Text S1. Methods for RNA extraction to transcriptome assembly.**
